# Anatabine ameliorates intestinal inflammation and reduces the production of pro-inflammatory factors in a dextran sulfate sodium mouse model of colitis

**DOI:** 10.1186/s12950-020-00260-6

**Published:** 2020-08-24

**Authors:** Pedro A. Ruiz Castro, Ulrike Kogel, Giuseppe Lo Sasso, Blaine W. Phillips, Alain Sewer, Bjorn Titz, Llenalia Garcia, Athanasios Kondylis, Emmanuel Guedj, Dariusz Peric, David Bornand, Remi Dulize, Celine Merg, Maica Corciulo, Nikolai V. Ivanov, Manuel C. Peitsch, Julia Hoeng

**Affiliations:** 1 Philip Morris International R&D, Philip Morris Products S.A, Quai Jeanrenaud 5, 2000 Neuchâtel, Switzerland; 2 Philip Morris International Research Laboratories Pte Ltd, 50 Science Park Road, The Kendall #02-07, Science Park II, Singapore, 117406 Singapore

**Keywords:** Plant-derived alkaloid, Mouse model, Nicotine, Colitis

## Abstract

**Background:**

Inflammatory bowel disease (IBD) is the collective term for chronic immune-mediated diseases of unknown, multifactorial etiology, arising from the interplay between genetic and environmental factors and including two main disease manifestations: ulcerative colitis (UC) and Crohn’s disease. In the last few decades, naturally occurring alkaloids have gained interest because of their substantial anti-inflammatory effects in several animal models of disease. Studies on mouse models of IBD have demonstrated the anti-inflammatory action of the main tobacco alkaloid, nicotine. In addition, anatabine, a minor tobacco alkaloid also present in peppers, tomato, and eggplant presents anti-inflammatory properties in vivo and in vitro. In this study, we aimed to evaluate the anti-inflammatory properties of nicotine and anatabine in a dextran sulfate sodium (DSS) mouse model of UC.

**Results:**

Oral administration of anatabine, but not nicotine, reduced the clinical symptoms of DSS-induced colitis. The result of gene expression analysis suggested that anatabine had a restorative effect on global DSS-induced gene expression profiles, while nicotine only had limited effects. Accordingly, MAP findings revealed that anatabine reduced the colonic abundance of DSS-associated cytokines and increased IL-10 abundance.

**Conclusions:**

Our results support the amelioration of inflammatory effects by anatabine in the DSS mouse model of UC, and suggest that anatabine constitutes a promising therapeutic agent for IBD treatment.

## Background

Crohn’s disease (CD) and ulcerative colitis (UC), the main clinical phenotypes of inflammatory bowel diseases (IBD), are chronic relapsing inflammatory disorders that affect the gastrointestinal tract. IBD are thought to result from an inappropriate and continuing inflammatory response to commensal microbes in a genetically susceptible host. Environmental triggers such as increased hygiene, drug use, stress, smoking, and diet influence the onset of the disease [1]. Over the past decades, naturally occurring alkaloids from plant or medicinal herb sources have sparked considerable interest because of their significant anti-inflammatory and antioxidant properties [2–4].

Alkaloids—a class of natural bioactive compounds derived from amino acids that contain one or more heterocyclic nitrogen atoms—are produced by a wide range of living organisms, such as bacteria, fungi, plants, and animals [5]. In plants, alkaloids are produced as secondary metabolites in response to environmental biotic or abiotic interactions, and they confer protection through a range of insecticidal, antimicrobial and pharmacological attributes. The anti-inflammatory activities of plant-derived alkaloids have been documented in several animal models of disease, including respiratory distress [6], spinal cord injury [7], hepatic fibrosis [8], cancer [9], and IBD [10, 11]. The protective effects of alkaloids have been attributed to amelioration of inflammatory responses and colonic oxidative stress [12, 13], promotion of epithelial barrier function [14], and positive regulation of gut microbiota [15].

Pyridine alkaloids present in tobacco (*Nicotiana tabacum*) have been the subject of intensive research. Nicotine, the major alkaloid in tobacco, accounts for approximately 95% of the total alkaloid content of tobacco, while the structurally related nornicotine and anatabine are the most abundant minor pyridine alkaloids, accounting for 4 to 5% of total alkaloids [16]. Other pyridine alkaloids in tobacco, such as anabasine, anabaseine, and cotinine, are present in smaller amounts [17]. Nicotine and all minor tobacco alkaloids have been shown to be pharmacologically active upon binding to several nicotinic acetylcholine receptors (nAChRs) [18]. Tobacco nAChR agonists such as nicotine, anatabine, anabasine, anabaseine, and cotinine display protective effects in animal models of several inflammatory conditions, including sepsis [19], Parkinson’s disease [20], Alzheimer’s disease [21], and IBD [22].

Several in vitro and in vivo studies have shown a clear nicotine-dependent positive effect on inflammatory processes [23, 24]. In a previous study, oral nicotine administration reduced the severity of DSS-induced colitis and reduced colonic TNFα synthesis, while subcutaneous injection or minipump infusion had no effect, highlighting the crucial role of administration route for the protective effects of nicotine in DSS colitis [25]. Nicotine has also been shown to attenuate the severity of DSS colitis and expression of IL-6 in CD4T cells [23]. A recent study suggested that nicotine ameliorates DSS-induced inflammation through inhibition of signal transducer and activator of transcription (STAT)3 in gut-infiltrated lymphocytes and intestinal epithelial cells [26]. Recently, nicotine was shown to cause a decrease in leukocyte recruitment, disease activity index (DAI), and histological score in DSS colitis and block TNF-mediated expression of mucosal vascular addresin cell adhesion molecule-1 in endothelial cells. These authors concluded that nicotine suppresses inflammation through downregulation of adhesion molecules in the gut [27]. Nonetheless, clinical studies on the efficacy and tolerability of nicotine have shown that therapeutic application of nicotine for treatment of UC is limited because of frequent adverse effects and nicotine inconsistent efficacy in maintaining remission in UC patients [28, 29].

Anatabine is found in plants of the Solanacea family, which includes tobacco, peppers, tomato, and eggplant [30]. Little is known about the biological properties of anatabine, although several studies have suggested that this alkaloid is a potential candidate compound for anti-inflammatory drug development [21, 31]. Anatabine was marketed in the US as a dietary supplement under the name Anatabloc. In an internet-based survey, approximately 90% of all users rated the effect of anatabine supplementation as good or excellent for joint pain, stiffness, and functionality [32]. Anatabine has been shown to inhibit lipopolysaccharide (LPS)-induced pro-inflammatory gene expression as well as NF-κB and STAT3 phosphorylation in human neuroblastoma SH-SY5Y, HEK293, human microglia, and human blood mononuclear cells [33] as well as in the brain and spleen of mouse models of autoimmune encephalomyelitis [31] and Alzheimer’s disease [33]. In SH-SY5Y cells, anatabine also reduced the expression of beta-secretase 1—the rate limiting enzyme for β-amyloid peptide production, which is a major hallmark of Alzheimer’s disease—through inhibition of NF-κB activation [21].

In this study, we aimed to assess the anti-inflammatory effects of the tobacco alkaloids nicotine and anatabine in the established DSS mouse model of UC [34]. Our results show that oral administration of anatabine, but not nicotine, ameliorates the clinical manifestations of DSS treatment in mice. The results of gene expression analysis indicated that anatabine had a partial restorative effect on global DSS-induced gene expression profiles, while nicotine only had minimal effects. Moreover, multi-analyte profiling (MAP) showed that anatabine, but not nicotine, suppresses the production of IL-6, IL-1α, TNFα, granulocyte-colony stimulating factor (G-CSF), and keratinocyte chemoattractant (KC) while increasing IL-10 expression in the colon of DSS-treated mice. For an overview of the study concept and analytical procedures, see “Online Resource 1”.

## Results

### Anatabine has a protective effect in the DSS mouse model of colitis

**Fig. 1:**
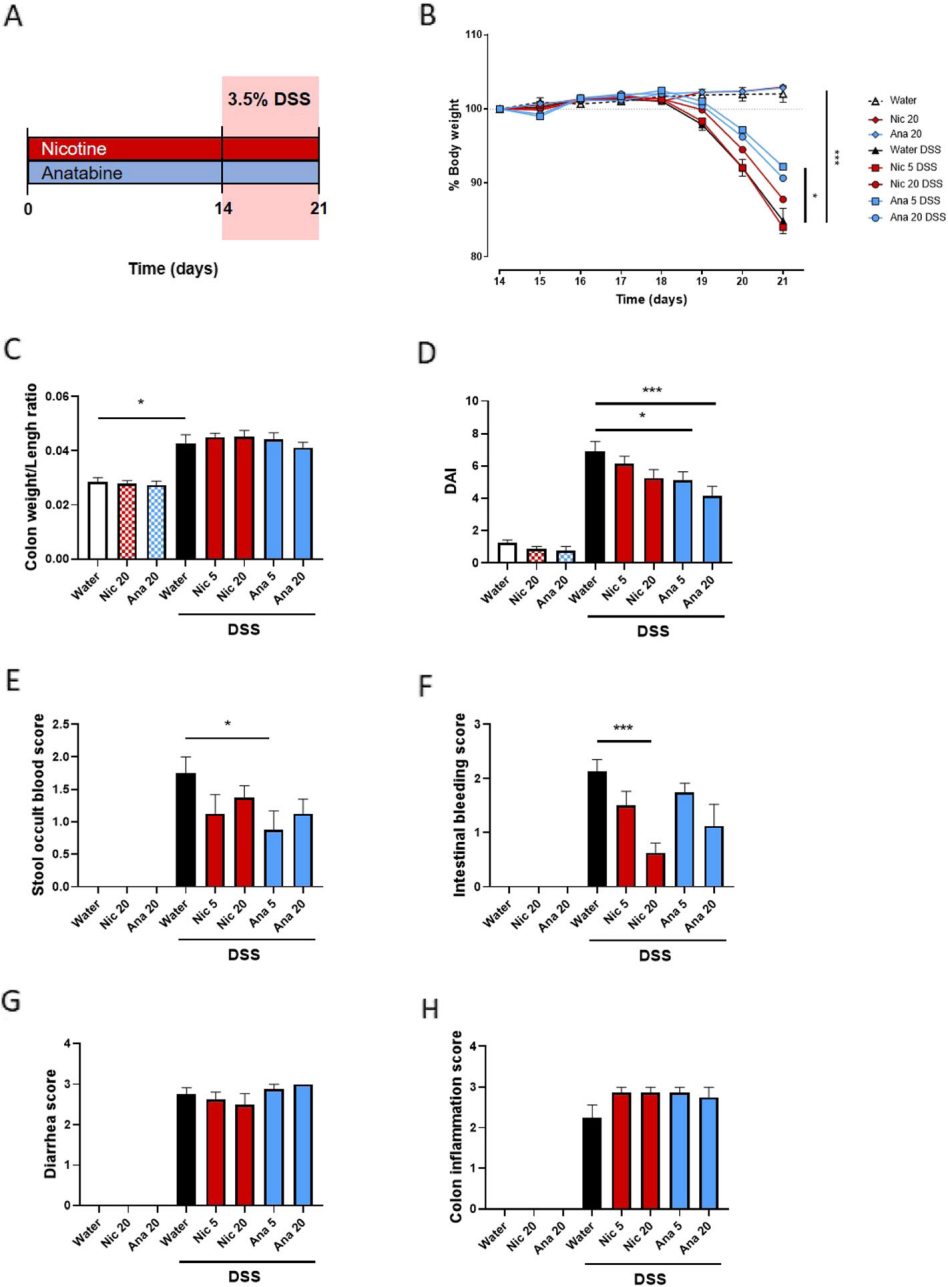
Clinical parameters of DSS-treated mice administered nicotine or anatabine. **a** Mice were orally administered nicotine or anatabine (5 or 20 mg/kg) for a total of 21 days. Colitis was induced by oral administration of 3.5% DSS in drinking water ad libitum during days 14–21. **b** Body weight changes in mice during the colitis induction and recovery phases. Body weight changes were calculated as percentage relative to the starting day of DSS treatment (day 0). **c** Colon weight/length ratio are represented as mg/cm of colon. **d** DAI was calculated according to weight loss, colon weight/length ratio, and intestinal bleeding. **e** Stool occult blood score. **f** Intestinal bleeding score. **g** Diarrhea score. **h** Colon inflammatory status. Data are shown as mean ± SEM; *p < 0.05; ***p < 0.001. Nic 5, nicotine 5 mg/kg; Nic 20, nicotine 20 mg/kg; Ana 5, anatabine 5 mg/kg; Ana 20, anatabine 20 mg/kg

To study the anti-inflammatory properties of nicotine and anatabine, C57BL/6 mice were provided with nicotine or anatabine in drinking water for a total of 21 days. Colitis was induced by oral administration of 3.5% DSS in drinking water ad libitum during days 14–21 (Fig. 1a). DSS-treated mice developed colitis, as evident from the body weight loss (Fig. 1b), increased colon weight/length ratio (Fig. 1c), increased DAI (Fig. 1d), increased stool occult blood score (Fig. 1e), increased intestinal bleeding score (Fig. 1f), increased diarrhea score (Fig. 1g), and increased colon inflammation score (Fig. 1h). In mice not subjected to DSS treatment, nicotine and anatabine administration had no significant effect on these parameters. However, mice treated concomitantly with anatabine and DSS showed a decrease in colitis severity. In particular, in mice that received concomitant anatabine and DSS treatment, anatabine improved body weight recovery at 5 mg/kg (*p* < 0.05) (Fig. 1b), caused a decrease in global DAI relative to that in DSS-treated mice at daily dose of 5 (*p* < 0.05) and 20 mg/kg (*p* < 0.001) (Fig. 1c), and reduced the stool occult blood score at 5 mg/kg (*p* < 0.05) (Fig. 1e). Interestingly, nicotine but not anatabine improved the intestinal bleeding score relative to that in DSS-administered mice at 20 mg/kg (*p* < 0.001) (Fig. 1f). The diarrhea score (Fig. 1g) and the colon inflammation score (Fig. 1h) were not affected by nicotine or anatabine. No variation in water consumption was registered across the different experimental groups (Online Resource 2).

### Anatabine reduces DSS-induced gene expression changes in the distal colon on a global level

**Fig. 2:**
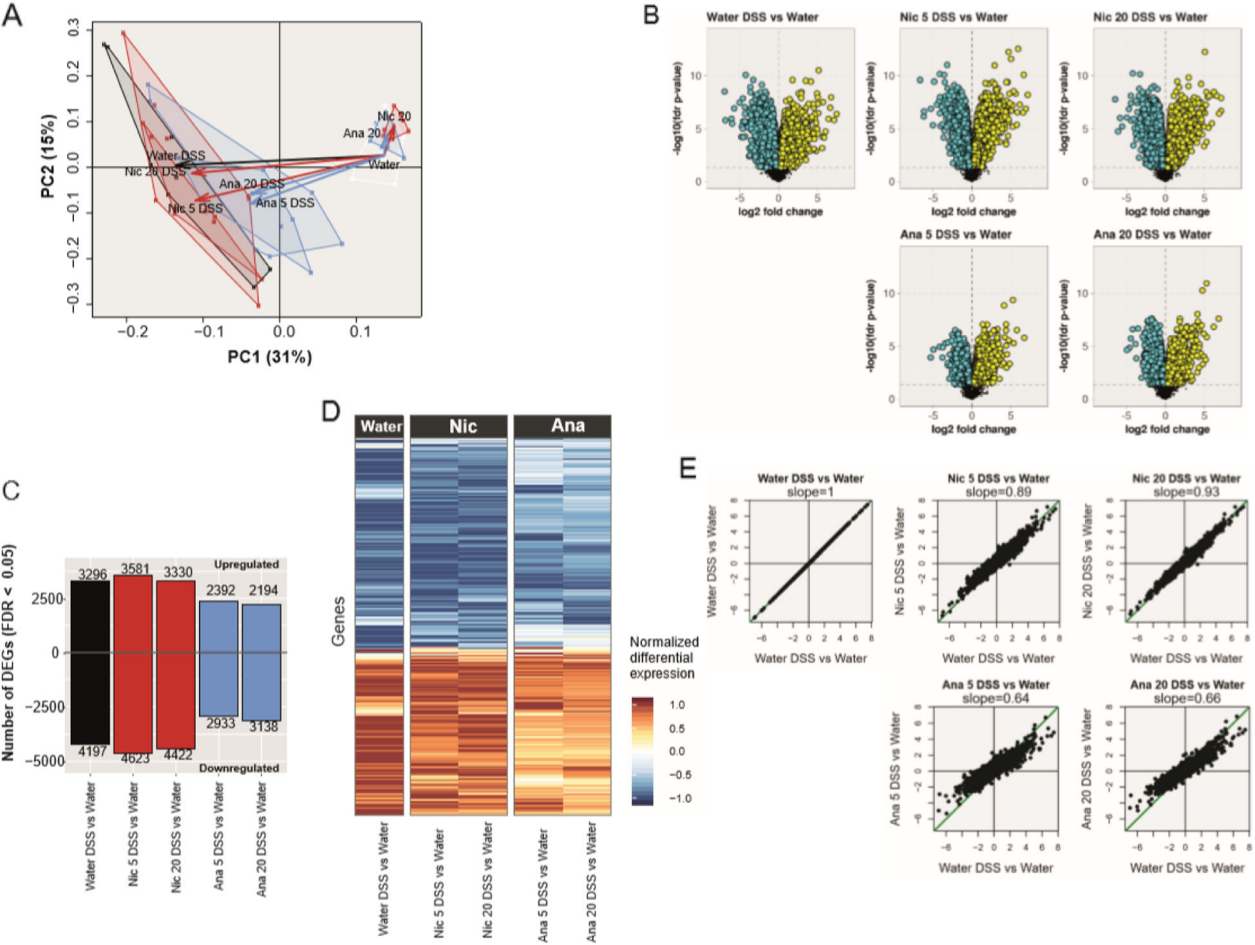
Transcriptomics results of colon biopsy samples. **a** Principal component (PC) analysis. The plot displays all samples in the reduced PC1–PC2 plane covering 31% + 15% = 46% of the total data variance. It allows us to examine the relationships between the various groups and treatments. Remarkably, PC1 captured the (pure) DSS effect (black arrow), while PC2 captured the (pure) exposure effects of anatabine and nicotine (blue and red arrows, respectively). **b** Volcano plots for individual gene differential expressions. The horizontal axis represents the log2 differential expression (“fold changes”), and the vertical axis represents its statistical significance as -log10 FDR. **c** Number of differentially expressed genes. For the selected pairwise comparisons, the bar plot displays the number of genes with positive or negative fold changes with corresponding statistically significant FDR ≤ 0.05. Note that the lower FDR values observed for the “Ana 5/20 DSS” comparisons do not necessarily prefigure a reduction of biological effects, because the statistics underlying the FDR/*p* values also depend on the gene expression variance within the experimental groups. **d** High-level heatmap of the statistically significant differentially expressed genes for the selected pairwise comparisons. In order to provide a comprehensible display of the large (9728 × 5) fold-change matrix, we first normalized its rows to their maximum absolute values and then reordered them by hierarchical clustering (complete linkage method) on the basis of their Euclidean distances. **e** Scatter plot from the comparisons between the pure and exposure-modified DSS effects by using the differential gene expression results. The horizontal axis of the scatter plots represents the fold changes of the pure DSS effect (“Water DSS vs Water” pairwise comparison), whereas the vertical axis contains the corresponding values in case of anatabine or nicotine exposure (“anatabine/nicotine 5/20 DSS vs Water” pairwise comparisons). In an ideal case (included as a reference [first plot], the best-fitted straight line coincides with the diagonal (indicated in green), and its slope is equal to 1. The transcriptomics effects of anatabine or nicotine exposure were quantified by the slope of the best-fitted straight lines indicated on each plot. Nic 5, nicotine 5 mg/kg; Nic 20, nicotine 20 mg/kg; Ana 5, anatabine 5 mg/kg; Ana 20, anatabine 20 mg/kg

To complement these pathological findings, we analyzed the colon transcriptome. DSS-elicited lesions in most mouse inbred strains, including C57BL/6 mice, have been shown to be more pronounced in the distal colon [35, 36], therefore we focused our study on that portion of the large intestine. Principal component analysis clearly captured the effect of DSS treatment on the first principal component (31% explained variance) (Fig. 2a). While nicotine treatment did not produce a pronounced effect on the first principal component in the DSS-treatment context, anatabine treatment in combination with DSS produced a response more similar to that observed in the water controls (no DSS), indicating that anatabine had an ameliorating effect on DSS-induced gene expression changes. The results of our differential gene expression analysis suggested substantial perturbation of the colon transcriptome upon DSS treatment (FDR < 0.05) (Fig. 2b–c). Application of DSS in drinking water caused significant changes in nearly 7500 genes in colon tissues. Addition of nicotine to DSS-containing drinking water slightly increased the number of differentially expressed genes. In contrast, addition of anatabine decreased the number of differentially expressed genes upon DSS treatment by approximately 30% (Fig. 2b and c). This alleviating effect of anatabine treatment on DSS-induced gene expression profiles was also apparent in the global gene expression heatmap, which showed a global reduction of expression fold changes upon anatabine treatment (Fig. 2d and e). Of note, in the absence of DSS, anatabine treatment did not result in differential expression of genes, and nicotine treatment alone had minor effects (FDR *p* value < 0.05; Online Resource 3).

### Anatabine reduces DSS-induced inflammatory gene expression in the distal colon

**Fig. 3:**
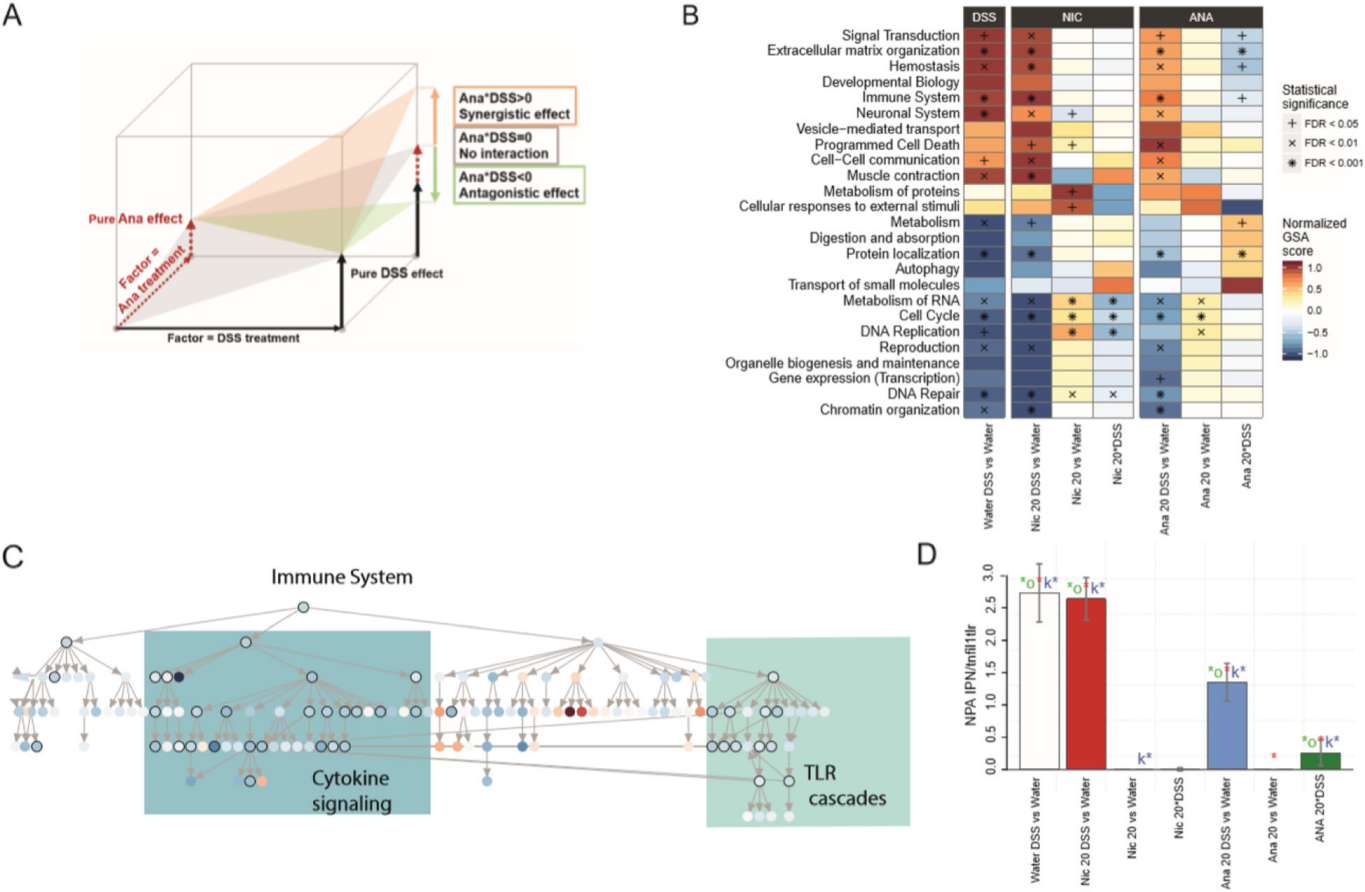
Biological interpretation of the transcriptomics results. **a** Schematic representation of the effects of anatabine/nicotine exposure as a modification of the pure DSS effect. The measured combined “anatabine/nicotine DSS” effect (captured by the pairwise comparisons “anatabine/nicotine DSS vs Water”) can be viewed as the sum of the pure effects of the two factors “DSS treatment” and “anatabine/nicotine exposure”, together with the adjusting two-factor interaction term “anatabine/nicotine*DSS”. Anatabine/nicotine exposure is synergistic with DSS treatment if the interaction term has the same sign as the pure DSS effect (e.g., both are positive, as in the schema); in contrast, the relationship is antagonistic if the interaction term has an opposite sign to the pure DSS effect. **b** GSA results for the top 25 Reactome pathway categories. The heatmap displays the GSA scores (normalized row-wise to their maximum absolute values) as well as their competitive Q1 statistical significance. The FDRs were calculated only among the top 25 Reactome pathway categories, which are sufficiently distinct in their gene content to assume independence of their enrichment results. **c** Hierarchical representation of the GSA results for all pathways contained in the top Reactome “Immune System” category and for the two-factor “Ana 20*DSS” interaction. The color map corresponds to the normalized GSA scores contained in the interval [− 1,1], whereas their statistical significance (competitive Q1 p values ≤0.05) is indicated by black circles around the nodes. The actual labels of the nodes (i.e., the Reactome pathway names) can be found in Online Resource 5. The tree-like structure of the Reactome pathway collection enables top-down investigation within the relevant pathway categories by identifying increasingly specific biological processes (i.e., involving fewer and fewer genes) along the longest paths connecting the statistically significant pathways. **d** NPA results for the TLR–IL1R–TNFR network model. The bar plot displays the NPA values for the selected contrasts, and their statistical significance is indicated by the three colored asterisks. Note that, by definition, the positive NPA values depend on the square of the input gene-level fold changes and, therefore, might amplify the differences between the contrasts without preserving the additive relationships among them. Nic 5, nicotine 5 mg/kg; Nic 20, nicotine 20 mg/kg; Ana 5, anatabine 5 mg/kg; Ana 20, anatabine 20 mg/kg

To gain a more mechanistic understanding of the effects of nicotine and anatabine treatment, we further investigated gene expression changes at the level of functional gene sets and a UC-relevant causal network model. GSA of the Reactome database showed changes across multiple functional categories (Fig. 3b). The effects on functional categories in DSS-treated mice administered nicotine appeared rather similar to those in mice treated with DSS alone, whereas administration of anatabine resulted in a general repression of DSS-mediated effects. We also evaluated the interaction terms between nicotine/anatabine and DSS treatment to more directly focus on the modulating effect of anatabine and nicotine treatment on DSS effects (Fig. 3a; Online Resource 4). The following six biological categories showed significant interaction terms upon anatabine and DSS treatment (Ana 20*DSS; FDR < 0.05), all supporting a significant suppression of DSS-induced effects in the presence of anatabine: “Immune System”, “Extracellular Matrix Organization”, “Protein Localization”, “Metabolism”, “Hemostasis”, and “Signal transduction” (Fig. 3b). Of these, we further explored “Immune System”, “Extracellular Matrix Organization”, and “Signal Transduction” as the most relevant categories in IBD.

The hierarchical organization of the Reactome database allowed us to investigate the underlying functional changes in more detail. The “Immune System” category includes “Adaptive Immune System”, “Cytokine Signaling in Immune System”, and “Innate Immune System”. In particular, within these immune categories, “Cytokine Signaling” (e.g., including IL-6 family signaling) and “Toll-like Receptor Cascades” were modulated with significant interaction terms (*p* < 0.05) (Fig. 3c; Online Resource 5; Online Resource 12). Within the Reactome “Signal Transduction” category “Signaling by Receptor Tyrosine Kinases”, “Signaling by Rho GTPases”, “Signaling by Transforming Growth Factor (TGF)-Beta Family Members”, “MAPK Family Signaling Cascades”, “Integrin Signaling”, “Signaling by Erythropoietin”, and “Signaling by GPCR” were found to be significantly impacted (Online Resource 6; Online Resource 12). Within the “Extracellular Matrix Organization” category, almost all subcategories were perturbed, including “Degradation of the Extracellular Matrix”, “Elastic Fiber Formation”, and “Extracellular Matrix Proteoglycans” (Online Resource 7; Online Resource 12).

“Degradation of the Extracellular Matrix” includes biological processes such as activation of matrix metalloproteinases (MMPs) and collagen degradation.

To further follow up on the effects of nicotine and anatabine on inflammatory signaling, we evaluated the perturbation of the UC-relevant TLR/IL1R/TNFR network model [37]. By using an established network enrichment approach [37], we inferred the (activation) states of the network nodes on the basis of the observed gene expression changes and calculated the overall network perturbation amplitude for each group comparison (Fig. 3c). DSS treatment had a strong activating effect on this signaling network, including activation of several core signaling nodes such as IL-1R-associated kinase (IRAK)1, IRAK4, and myeloid differentiation primary response 88 (MYD88) (Online Resource 11 “heatmap leading nodes”). Of note, the network perturbation induced by DSS treatment was reduced only in the presence of anatabine. With this, the results of network analysis further supported the ameliorating effect of anatabine on DSS-mediated inflammation, whereas no similar effect was apparent upon nicotine treatment.

**Fig. 4:**
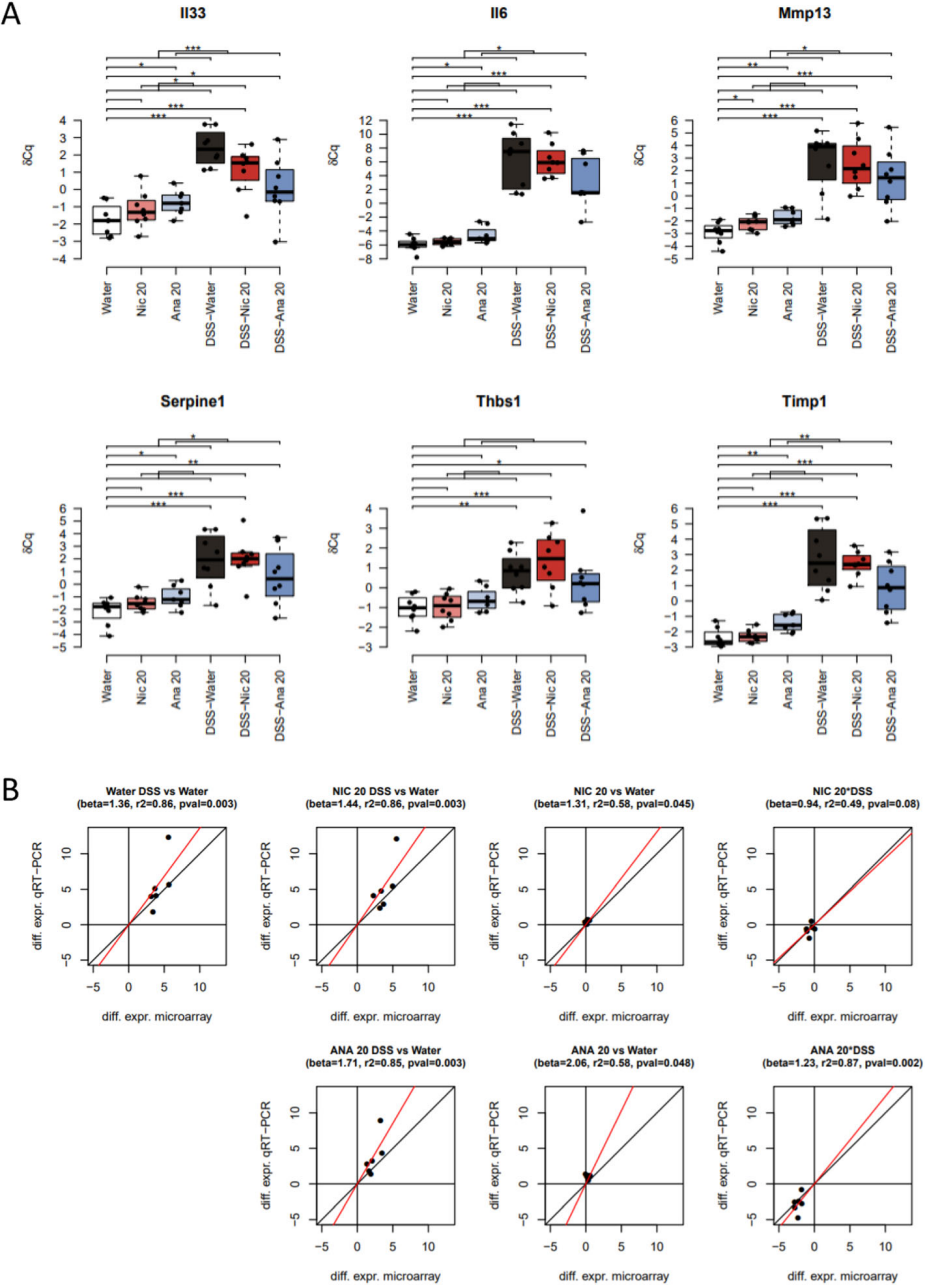
Validation of microarray transcriptomic results using qPCR. **a** Box-and-whisker plots for the distribution of the qPCR expression levels (*δCq*) of the selected genes. The boxes represent the quartiles while the whiskers extend to the most extreme data point which is no more than 1.5 times the interquartile range from the box. The horizontal brackets indicate the statistical significance of the corresponding comparisons (*,**,*** mean *p*-value smaller than 0.05, 0.01, 0.001, respectively). **b** Scatter plots comparing the mouse colon differential expression values obtained by microarray (horizontal axis) and qPCR (vertical axis). The following similarity metrics were indicated: “beta” is the slope of the best intercept-free linear fit between microarray and qPCR values, “r2” is the coefficient of determination measuring the “goodness-of-fit”, and “pval” is the p-value associated to the null hypothesis beta = 0. Nic 20, nicotine 20 mg/kg; Ana 20, anatabine 20 mg/kg

To validate the observations obtained by microarray transcriptomics, we quantified the expression of six “leading-edge” genes—genes of the gene set with the highest differential expression values—from the “Immune System”, “Extracellular Matrix Organization”, and “Signal Transduction” reactome categories using real-time quantitative PCR (qPCR). Selected genes included *Il33* (“Signal Transduction” and “Immune System” categories), *Il6* (“Signal Transduction” and “Immune System” categories), *Mmp13* (“Extracellular Matrix Organization” category), *Serpine1* (“Signal Transduction” and “Extracellular Matrix Organization” categories), *Thbs1* (thrombospondin 1; “Signal Transduction” and “Extracellular Matrix Organization” categories), and *Timp1* (tissue inhibitor of metalloproteinase; “Extracellular Matrix Organization” and “Immune System” categories). qPCR results showed a clear decrease in DSS-induced expression of *Il33, Il6, Mmp13, Serpine1, Thbs1*, and *Timp1* expression in the presence of anatabine at 20 mg/kg, thereby confirming the findings obtained from the microarray data (Fig. 4).

### Anatabine decreases DSS-induced pro-inflammatory cytokine production and promotes IL-10 expression

**Fig. 5:**
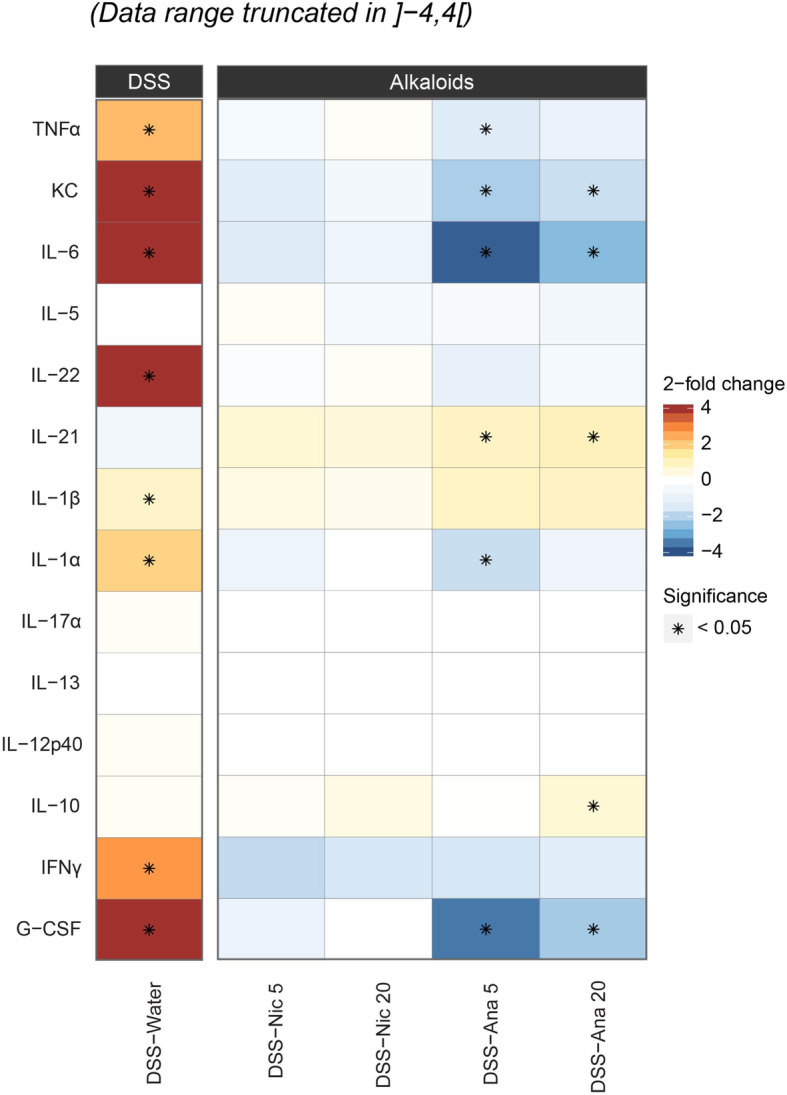
MAP results for colon biopsies. Statistical assessment of the differences observed in the abundance of selected cytokines between the study experimental groups and water control. Fold changes in the treatment groups (nicotine and anatabine at 5 and 20 mg/kg DSS) relative to water (control) are illustrated with colors ranging from blue (decrease) to red (increase). *, statistically significant differences on the basis of raw *p*-values < 0.05 (no adjustment has been made for multiple testing). Grey cells highlight missing estimates on the observed differences due to lack of cytokine quantifications. Nic 5, nicotine 5 mg/kg; Nic 20, nicotine 20 mg/kg; Ana 5, anatabine 5 mg/kg; Ana 20, anatabine 20 mg/kg

Next, we sought to evaluate the impact of nicotine and anatabine on the expression of colonic inflammatory cytokines by MAP. In line with the previous data, anatabine, but not nicotine, significantly reduced the abundance of DSS-associated inflammatory cytokines including IL-6, KC, TNFα, IL-1α, and G-CSF, whereas it increased the levels of the anti-inflammatory cytokine IL-10 at a daily dose of 20 mg/kg (Fig. 5). Interestingly, anatabine also increased the abundance of IL-21 and showed a clear tendency towards increasing colonic IL-1β levels (Fig. 5). Taken together, the MAP data confirm the anti-inflammatory effects of anatabine in DSS-induced colitis.

## Discussion

Our study shows that oral administration of anatabine, but not nicotine, reduces the clinical manifestations of DSS-induced colitis in a mouse model. In line with these findings, anatabine demonstrated a global downregulatory effect on DSS-induced gene expression changes in the colon, whereas the effects of nicotine were more limited. In particular, the results of gene expression profiling further supported the reduction of inflammatory signaling processes upon anatabine treatment, including suppression of IL-6 signaling (as shown by GSA findings) and TLR signaling (as shown by the results of network perturbation analysis and GSA). MAP also showed a significant decrease in the abundance of IL-6, KC, TNFα, IL-1α, and G-CSF and an increase in the expression of IL-21 and the anti-inflammatory cytokine IL-10.

Several studies have reported on the anti-inflammatory effects of nicotine on the DSS mouse model of UC [27]. Subcutaneous administration of nicotine was shown to ameliorate tissue injury in DSS colitis and IL-6 expression in CD4T cells via α7-nAChRs [23]. Nicotine was also shown to decrease the activation of STAT3 through induction of miR-124 in gut-infiltrated lymphocytes and intestinal epithelial cells [26]. Strikingly, AlSharari et al. observed that oral, but not subcutaneous injection of nicotine, ameliorated intestinal inflammation and colonic TNFα expression in DSS-treated mice [25]. In spite of the reported beneficial effects, our results do not support a protective effect of orally administered nicotine on DSS colitis. Of note, we found that nicotine significantly reduced DSS-associated intestinal bleeding, which was the only clinical parameter affected by this tobacco alkaloid. The vasoconstrictor effects of nicotine are well stablished [38]. In the gut mucosa, nicotine decreases blood flow [39], and cigarette smoking decreases rectal blood flow to normal levels in patients with UC [40]. However, how changes in blood flow affect the pathophysiology of UC is still unclear. The possible therapeutic use of nicotine to induce remission in UC patients has been evaluated in five clinical studies [41–45] and two meta-analyses [28, 46]. These studies have demonstrated a variable efficacy of nicotine therapy in induction of remission [28], with several studies showing no effect [41, 44]. Moreover, a high frequency of adverse events increased the withdrawal rate in the nicotine group in some studies, thus limiting the therapeutic benefit of nicotine [29].

To the best of our knowledge, the present study is the first to assess the impact of anatabine on experimental colitis. Gene expression analysis of distal colon biopsy specimens highlighted the anti-inflammatory properties of anatabine in multiple functional categories, including “Immune Responses”, “Signal Transduction”, and “Extracellular Matrix Organization”, which in turn encompassed several TLR and cytokine signaling pathways. Genes contributing to the downregulation of TLR cascades included TLR2, TLR4, and TLR6 and a number of downstream signaling factors shared by several TLRs, including MYD88, RIPK2, IRAK3, IRAK4, and NOD1 (nucleotide-binding oligomerization domain-containing protein 1), as well as the nuclear factors ELK1, Fos, cyclic adenosine monophosphate (cAMP) response element-binding protein (CREB)1, activating transcription factor (ATF)1, and ATF2 (See Online Resource of the respective gene lists). For the cytokine signaling pathways, the contributing genes included IL-6 and IL-6 receptor α, IFNγ, IL-4, CXCL10, IL-22 receptor α2, IL-2 receptor α, TNF receptor superfamily member 1A, IL-17α, and IL-17 receptor α, JAK1, JAK2, STAT3, and STAT4. Members of the NF-κB signaling pathway also contributed to the overall reduction in inflammatory cascades, including NF-κB p65, p105, and p100 subunits, IκBα, and the NF-κB activating protein TAB3. In agreement with the results of transcriptional analysis, MAP findings showed a significant decrease in DSS-associated IL-6, KC, TNFα, IL-1α, and G-CSF protein expression and an increase in IL-10 expression in the presence of anatabine. Strikingly, while TLR downstream factors modulated by anatabine are shared by most TLRs, the majority of cytokine-associated signaling molecules are specific for each pathway. The seemingly pleiotropic regulatory effects of anatabine suggest that this alkaloid reduces inflammation by inhibiting the expression of several factors involved in different pro-inflammatory signaling cascades.

Previous studies using in vitro and in vivo disease models have demonstrated the anti-inflammatory effects of anatabine [21, 31]. Paris et al. showed that this pyridine alkaloid reduced the plasma levels of IL-1β, IL-6, and IL-17A as well as the expression of IL-1β, INFγ, and TNFα in the spleen of experimental autoimmune encephalomyelitis mice [31]. These authors also showed that anatabine suppressed STAT3 and NF-κB phosphorylation in the spleen and brain of these mice [31]. Anatabine also prevented LPS- and TNFα-induced NF-κB and STAT3 phosphorylation in SH-SY5Y and HEK cells, human microglia, and human blood mononuclear cells [33]. Additionally, anatabine prevented LPS-induced IL-1β expression in human whole blood as well as IL-1β, IL-6, and TNFα production in the plasma, kidney, and spleen in the LPS mouse model [33]. Phosphorylated STAT3, TNFα, and IL-6 were also downregulated in the presence of anatabine in a transgenic mouse model of Alzheimer’s disease [33].

Our results on the effects of anatabine in the DSS mouse model of UC are also in line with the findings of a substantial number of studies demonstrating the protective effects of natural alkaloids in several animal models of colitis [13, 47]. Intraperitoneal administration of the minor tobacco alkaloid and nicotinic receptor agonist anabaseine was shown to reduce tissue damage, myeloperoxidase activity, and colonic TNFα expression in a TNBS mouse model of colitis [22]. These mice also showed reduced NF-κB activation in lamina propria mononuclear cells, while mice administered a nicotinic receptor antagonist presented worse colitis symptoms than those treated with TNBS alone [22]. The algae alkaloid caulerpin reduces DSS colitis by suppressing NF-κB activation and subsequently inhibiting the colonic production of TNFα, IFNγ, IL-6, IFNγ, and IL-17 [48]. Oral administration of berberine also ameliorates DSS-induced colitis and downregulates the expression of TNFα, IFNγ, KC, and IL-17 in colonic macrophages [49]. The plant-derived alkaloid *N*-methylcytisine and the tea alkaloid theophylline mitigate colitis by downregulating TNFα, IL-1β, and IL-6 expression in DSS and acetic acid models of colitis, respectively [50, 51]. Induction of the anti-inflammatory cytokine IL-10 in the presence of natural alkaloids has also been reported. Thus, indirubin ameliorates DSS-induced colitis by suppressing the expression of colonic TNFα, IFNγ, and IL-2 and upregulating IL-10 [52]. *Additionally, indole alkaloids caulerpin and isatin have been shown to increase the expression of IL*-*10 in DSS and TNBS models of IBD, respectively* [48, 53]*.* Of note, our results show an increase in the abundance of IL-21 and a tendency towards increase in colonic IL-1β levels in the presence of anatabine. Although IL-21 expression is increased in many chronic inflammatory disorders, genetic deficiency of IL-21 is associated with IBD, and inhibition of IL-21 in the early phases of some inflammatory disorders exacerbates disease development, suggesting the dual role of IL-21 in the control of immune responses [54]. IL-21 also promotes IL-22 expression in mucosal T-cells through a mechanism involving STAT3, retinoid-related orphan receptor γt, and aryl hydrocarbon receptor, thereby helping protect immunodeficient mice from DSS colitis [55]. Interestingly, IL-21 has been recently shown to induce IL-1β production in dendritic cells through a STAT3-dependent but NF-κB-independent mechanism, thereby suggesting a link between IL-21 and IL-1β [56].

Mounting evidence suggests that alkaloids—in particular isoquinoline alkaloids present in traditional medicine herbs—exert their anti-inflammatory effects through regulation of NF-κB and STAT3 signaling pathways. For example, sanguinarine and cavidine suppress the expression of NF-κB p65 subunit, thereby reducing colonic TNFα and IL-6 in acetic acid-induced colitis [2, 11]. In the TNBS mouse model of colitis, berberine reduces IFNγ, IL-1α, IL-6, IL-17, and TNFα expression in colonic tissues and sera and suppresses Th1 and Th17 cells through reduction of STAT1, STAT3, and NF-κB phosphorylation [57]. Oral administration of boldine attenuates DSS-induced colitis and reduces colonic phosphorylation of STAT3 and NF-κB p65 subunit as well as the expression of TNFα, IL-6, and IL-17 [58]. Demethyleneberberine, tetrandrine, and norisoboldine show protective effects in the DSS colitis model and reduce the levels of colonic IL-1β, TNFα, and phosphorylated NF-κB p65 subunit [10, 59, 60].

According to the present GSA findings, genes affected by anatabine in the Reactome category “signal transduction” encompassed numerous GPCR-associated factors, including several G protein subunits, adenylate cyclases, Rho guanine nucleotide exchange factors, factors involved in Ca^2+^-mediated events, and phosphoinositide 3-kinase (PI3K)-associated signaling factors. The latter included the ubiquitously expressed class I PI3K isoforms p110α and p110γ (also known as PIK3CA and PIK3CG, respectively), PI3K regulatory subunit (PIK3R)5, PIK3R6, and AKT3. Similarly, several other alkaloids have been shown to reduce PI3K signaling cascades in the DSS mouse model of colitis. For example, quinolizidine alkaloids oxymatrine and aloperine ameliorate DSS colitis by inhibiting the PI3K/Akt pathway and T-cell responses [61, 62]. Plant-derived alkaloids evodiamine and rutaecarpine inhibit corticosterone production by decreasing the activity of cAMP-related pathways in rat zona fasciculata–reticularis cells [63]. Sinomenine, an isoquinoline alkaloid which ameliorates colitis and inflammatory gene expression in several mouse models of IBD [64, 65], also reduces the levels of cAMP, intracellular Ca^2+^, and phosphorylated CREB in morphine-treated SH-SY5Y cells [66]. Strikingly, our findings suggested that factors contributing to anatabine-associated reduction of GPCR cascades included a wide range of cyclic nucleotide phosphodiesterases (PDEs) —including several subtypes of PDE1, PDE3, PDE4, PDE7, PDE8, and PDE10—which modulate the intracellular concentrations of cAMP and cyclic guanosine monophosphate (cGMP) by regulating their rates of degradation [67]. In this regard, several naturally occurring alkaloids, such as caffeine and tolafentrine, also inhibit PDE activities [68, 69]. The opium alkaloid papaverine has been identified as a potent and specific inhibitor of PDE10A, with the ability to trigger cAMP and cGMP accumulation as well as CREB phosphorylation in several animal behavioral models [70, 71]. Taken together, our results suggest that anatabine exerts a dual effect on GPCR-associated pathways hindering the expression of GPCR-associated factors, while enhancing the accumulation of intracellular cAMP through reduction of PDE expression levels. Further research is needed to *understand* the overall impact of anatabine on GPCR-mediated responses.

Interestingly, in the present study, anatabine reduced the expression of factors in the Reactome category “Extracellular Matrix Organization”. Genes contributing to this reduction encompassed a wide range of factors involved in ECM deposition, including fibronectin 1, fibulin (FBLN)1, FBLN2, elastin, laminin, lysyl oxidases *(collagen* cross-linking enzymes), and fibrillar, network-forming, transmembrane, and fibril-associated collagens. Likewise, we observed a tendency towards reduced expression of a number of genes involved in ECM protein degradation, including several disintegrins and metalloproteinases with thrombospondin motifs, MMPs (including MMP8; MMP14; the gelatinases MMP2 and MMP9; and the stromelysins MMP3, MMP7, MMP10, and MMP12), and TIMP1 and TIMP2. Gene expression analysis identified factors involved in fibroblast proliferation and activation, including vimentin, various members of the TGFβ family, fibroblast growth factors, and fibroblast growth factor receptor (FGFR)1, FGFR2, and FGFR3. Overall, our data point to the possibility that the anti-inflammatory effects of anatabine span the regulation of IBD-associated fibrogenic responses, including fibroblast activation, ECM protein deposition, and breakdown of ECM proteins. A previous study highlighted the protective effects of intraperitoneally administered pentoxifylline—a xanthine alkaloid derived from the seeds of the cacao tree (*Theobroma cacao*)—on TNBS colitis in rats through limiting TGFβ1 accumulation and MMP3 and MMP9 activation [12]. Because wound-healing responses follow tissue injury and inflammation, it is plausible that the downregulation of extracellular matrix organization factors observed in the presence of anatabine is a consequence of reduced DSS inflammation rather than a direct effect of anatabine [72].

DSS induces an UC-like pathology through the breakdown of the gut epithelium [34]. Loss of epithelial barrier function in IBD is caused by enhanced apoptosis of epithelial cells and alterations in the architecture of epithelial tight junctions. Our results show that anatabine regulates the production of several pro-inflammatory cytokines involved in barrier disruption, including TNFα. TNFα influences the expression and the localization of tight junction proteins, thereby contributing to a leaky gut barrier [73] and anti-TNFα therapies have been shown to reduce intestinal epithelial cell apoptosis in IBD patients [74]. Some of the primary factors affected by anatabine are associated with GPCR signaling, which has been shown to regulate tight junction sealing. Thus, Gα_12/13_ triggers the dissociation of occludin and claudin-1 from the tight junction complex through the phosphorylation of zonula occludens (ZO)-1 and ZO-2, thereby increasing the paracellular permeability of the epithelial barrier [75]. Taken together, our results point to that part of the protective effects on DSS-colitis observed in the presence of anatabine could be ascribed to the downregulation of factors promoting the breakdown of the intestinal epithelial barrier.

An important limitation of our study is that it is not possible to discriminate between the individual contributions of the different cell types in intestinal mucosa to the overall anti-inflammatory response triggered by anatabine. Further research is needed to elucidate whether anatabine-mediated changes affect primarily one cell population, which in turn influences other cell types, or whether anatabine has synergistic or antagonistic effects in different cell types.

Our computational transcriptomic analysis allowed us to identify potential mechanism of anatabine in a highly un-targeted manner. This approach gave us the opportunity to discover multiple signaling pathways concurrently targeted by anatabine that are important for the development of IBD instead of focusing on one single signaling cascade. The pleiotropic effects observed in many natural bioactive compounds in general and anatabine in particular allows them to avoid the development of resistance due to the activation of alternative pathways, an issue observed with most single-target drugs [76]. Our study paves the way for further translational studies focusing on IBD-relevant molecular mechanisms modulated by anatabine.

In summary, our results show that oral administration of anatabine, but not nicotine, ameliorates the clinical symptoms of DSS colitis and regulates the expression of several factors involved in inflammatory and immune responses in the DSS mouse model of UC. Our data suggest that anatabine is a promising therapeutic agent for IBD treatment.

## Conclusions

Oral administration of anatabine ameliorated the clinical manifestations of DSS-treated mice and had a global regulatory effect in the expression of DSS-associated inflammatory and immune gene expression in the colon of these mice. Accordingly, anatabine induced a significant decrease in the abundance of pro-inflammatory cytokines, and reduced the protein expression of the anti-inflammatory cytokine IL-10 in the colon of DSS-treated mice. These results suggest that anatabine administration might constitute a promising approach for the treatment of IBD.

## Methods

### Animals

All procedures involving animals were performed in the A*STAR BCR facility, which is accredited by the Association for Assessment and Accreditation of Laboratory Animal Care International and licensed by the Agri-Food & Veterinary Authority of Singapore, with approval from an Institutional Animal Care and Use Committee and in compliance with the National Advisory Committee for Laboratory Animal Research Guidelines on the Care and Use of Animals for Scientific Purposes [77]. Male C57BL/6 mice (6–8 weeks of age) were obtained from InVivos Pte Ltd. (Singapore) and housed under specific hygienic conditions in filtered, conditioned fresh air at 21 ± 1 °C and 55 ± 5% humidity on a 12-h light–dark cycle. Male mice were used in this study since male mice have been shown to be more severely affected by DSS administration in the colon than females [36]. A gamma-irradiated pellet diet (Altromin 1324) and filtered tap water was provided ad libitum. The mice were allocated to their respective treatment groups (10 groups with 8 animals each) on the basis of body weight 3 days prior to the experimental start. The animals were randomized such that the mean body weight of the groups on day 3 was as close as possible, and the % standard deviation over mean body weight was well below 10% per group.

### Study design and DSS-induced colitis

Nicotine (Sigma-Aldrich, St. Louis, MO, USA) and anatabine citrate (Peakdale Molecular Ltd., Manchester, UK) were freshly prepared in filtered drinking water and provided to the animals on a daily basis for a total of 21 days targeting daily dosage of 5 or 20 mg/kg for the low and high treatment groups, respectively. The drinking water concentrations were 20 μg/ml and 80 μg/ml for nicotine (low and high) and 44 μg/ml and 88 μg/ml for anatabine (low and high). Colitis was induced by oral administration of DSS (TdB Consultancy, Uppsala, Sweden) through drinking water, which was supplied ad libitum during days 14–21 (Fig. 1a). DSS (molecular weight, 40,000 g/mol) was freshly prepared at a concentration of 3.5% in autoclaved sterile drinking water and provided to the animals on a daily basis. Control groups (no DSS) received drinking water from the same source. The general condition and health of the mice were monitored through routine body weight measurement and observation schedules. As a humane endpoint, the action planned in case an animal lost more than 20% of its body weight (relative to the weight at the start of DSS treatment) was to euthanize it. None of the animals used in this study reached a humane endpoint. Water consumption was recorded daily by weighing the water bottles (fresh and used bottles).

### Assessment of colitis

Body weight was recorded daily. On days 0 and 14, stool samples were collected and evaluated for occult blood by using Hemoccult-II® SENSA® elite test strips (Beckman Coulter, Brea, CA, USA). Scoring was conducted in accordance with the manufacturer’s procedures. Necropsy was performed on day 21. The animals were anaesthetized by intraperitoneal administration of 5% isoflurane. The colon was excised, and its length was determined for each animal. The colon was weighed after removal of its contents and then partitioned for molecular analysis. DAI was calculated according to weight loss, colon weight/length ratio, and intestinal bleeding.

### RNA isolation and transcriptomic data generation

The severity of DSS-induced injuries increases from the proximal to the distal colon in C57BL/6 mice. Consequently, we focused our analysis on this section of the intestine. RNA was extracted from distal colon tissues by using the miRNeasy mini kit protocol (Qiagen, Hilden, Germany) and further processed by using the IVT PLUS protocol (Affymetrix, Santa Clara, CA, USA). All processed RNA samples were hybridized on Affymetrix GeneChip® Mouse Genome 430 2.0 Arrays. CEL files were processed by using software packages from the Bioconductor suite of microarray analysis tools for the R statistical software environment [78]. Arrays that passed the quality controls (QC) were background corrected and normalized by the frozen robust multiarray method to generate the expression values [79]. The QC metrics—obtained using the affyPLM package—examined the distribution of log intensities, normalized unscaled standard error, relative log expression, and median absolute value relative log expression as well as the general aspect of the array pseudo- and raw images [80]. Additionally, the Mouse4302_Mm_ENTREZG v16.0 Brainarray Custom CDF environment was used for probe set-level summary to obtain the expression data matrix [81]. The array data have been deposited in the ArrayExpress public repository (E-MTAB-8543).

### Transcriptomic data analysis

#### Statistical models for differential gene expression

Given the experimental design involving two distinct factors (DSS induction and nicotine/anatabine exposure), we used several pairwise comparisons and two-factor statistical models to capture the various pure and combined effects, as well as their mutual influences. In this context, the interaction term from the two-factor model “1 + exposure + DSS + exposure * DSS” can be equivalently viewed as a difference of differences between the corresponding effects, that is, “exposure * DSS = ([exposure + DSS] vs [sham + DSS]) vs ([exposure] vs [sham]), where “sham” is the control level of the exposure factor. This interaction, therefore, captures the DSS-specific effect of the exposure, that is, the part of the changes induced by exposure to nicotine/anatabine that only appears in the context of DSS induction. The exposure * DSS interaction effect globally adds up to the pure exposure effect (in absence of DSS) and, when it is non-zero, can be synergistic (same direction as the pure effect) or antagonistic (opposed direction to the pure effect) (see also Fig. 3a). The differential gene expression values were calculated by applying the above statistical models to the normalized transcriptomic data by using the Bioconductor package limma [82]. The p - values for each contrast (pairwise comparison or term of the two-factor statistical models) were adjusted for multiple testing across genes by using the Benjamini–Hochberg false discovery rate (FDR) method [83]. Differentially expressed genes were defined as genes with FDR < 0.05 for a given contrast.

#### Gene-set analysis

We performed gene-set analysis (GSA) to interpret the results of differential gene expression in the appropriate biological context. As GSA examines the collective behavior of all genes involved in a common biological process, it is more sensitive than the over-representation methods that only use differentially expressed genes [84]. We determined the statistical significance of gene-set enrichment by using suitable competitive Q1 enrichment statistics, which were calculated by using the CAMERA method implemented in the limma package [85]. Because of the potentially strong dependences between gene sets (see hereafter), we decided not to apply multiple testing corrections to the obtained enrichment *p*-values. We used Reactome pathways as the collection of gene sets [86]. Reactome was selected as an appropriate open-access gene-set collection because of its comprehensive coverage of immune-related pathways (1643 gene sets in total, 187 of which are immune system-related). In order to leverage the particular structure of the pathways contained in Reactome, we used the available hierarchical relationships to group them into 25 top categories. As the resulting pathway graphs are not strictly trees (but still very close to them), we applied the Reingold–Tilford layout function from the igraph package to find the best approximative hierarchical trees for each top category [87].

#### Network perturbation amplitude

The network perturbation amplitude (NPA) method quantifies the overall treatment-induced perturbation of the biological mechanisms described by the causal network model chosen a priori and has previously been applied successfully in the context of IBD [37]. Instead of assuming that differential gene expression necessarily results in a change in the translated protein abundance or activity, the NPA approach follows reverse-causal reasoning, which infers the changes in the activity of a network node entity from the differential expression of the genes known to be collectively regulated by this entity [37, 85]. Unlike GSA, which uses competitive Q1 enrichment statistics for determining the most biologically relevant pathways for one given contrast, NPA enables comparison of the relevance of the considered network model across multiple contrasts. In the study cited above, the dedicated toll-like receptor (TLR)–IL-1 receptor (IL1R)–TNF receptor (TNFR) network model was used to contextualize the effects captured by the various contrasts in the case of the core inflammatory cascades.

### Gene expression quantification by real-time quantitative PCR (qPCR)

For reverse transcription, the RT^2^ First Strand Kit (Qiagen, Hilden, Germany), was used following the manufacturer’s instructions. Genomic DNA contamination was evaluated with RT^2^ qPCR Primer gDNA Control kit (Qiagen). qPCR was carried out in a QuantStudio 5 Real-Time PCR System (ThermoFisher, MA, USA), according to the manufacturer’s instructions, under the following cycling conditions: 2 min at 95 °C, followed by 40 cycles of 95 °C for 1 s and 60 °C for 20 s with the TaqMan Fast Universal Mastermix (ThermoFisher). TaqMan Gene Expression Assays (all from ThermoFisher) used in this study were mouse *Il33*, *Il6, Mmp13, Serpine1*, *Thbs1*, *Timp1*, *Actb* (β-actin), *B2m* (β-2 microglobulin), and *Gapdh* (Glyceraldehyde-3-phosphate dehydrogenase) (Table 1). *Cq* values generated with the QuantStudio Analysis Software Version 1.5.1 were reported as arithmetic means of three technical replicates and imported into the R statistical software environment for final processing. Using the three housekeeping genes (*Actb*, *B2m*, and *Gapdh*), the expression levels (*δCq*) were calculated by the mean-based normalization implemented in the Bioconductor NormqPCR package [88]. The differential expressions were obtained using the same statistical models as in transcriptomics (pairwise comparisons and “difference of differences” contrasts, see subsection “*Statistical models for differential gene expression”* above), with the difference that t-statistics was used for calculating the *p*-values (instead of moderated t-statistics). During the data processing, we observed clear interferences between the DSS treatment and the qPCR technique, an effect that has been already reported in the literature [89]. A principal component analysis of the *Cq* values of the three DSS-non-responding housekeeping genes *Actb*, *B2m*, and *Gapdh* revealed that the additional variance induced by the DSS-qPCR interference effect was uniformly distributed among them with weights approaching the ideal value of 1/√3 (with a relative difference < 0.5%). This observation confirmed the choice of applying mean-based normalization based on these housekeeping genes to optimally correct the qPCR data for this unwanted effect and to obtain reliable *δCq* values for all the tested genes.

**Table 1:** TaqMan gene expression assays

Gene	Full name	TaqMan assay ID	Reactome category
*Il33*	Interleukin 33	Mm00505403_m1	Signal Transduction Immune System
*Il6*	Interleukin 6	Mm00446190_m1	Signal Transduction Immune System
*Mmp13*	Matrix metallopeptidase 13	Mm00439491_m1	Extracellular Matrix Organization
*Serpine1*	Serine (or cysteine) peptidase inhibitor; clade E; member 1	Mm00435858_m1	Signal Transduction Extracellular Matrix Organization
*Thbs1*	Thrombospondin 1	Mm00449032_g1	Signal Transduction Extracellular Matrix Organization
*Timp1*	Tissue inhibitor of metalloproteinase 1	Mm01341361_m1	Extracellular Matrix Organization Immune System
*Gapdh*	Glyceraldehyde-3-phosphate dehydrogenase	Mm99999915_g1	Housekeeping gene
*B2m*	Beta-2 microglobulin	Mm00437762_m1	Housekeeping gene
*Actb*	Actin; beta	Mm04394036_g1	Housekeeping gene

### MAP of colon cytokines

We analyzed the abundance of inflammatory biomarkers in distal colon tissue homogenates. Dissected tissues were homogenized in 450 μL of homogenization buffer containing phosphate-buffered saline (Thermofisher, Reinach, Switzerland) supplemented with a complete miniprotease inhibitor cocktail (Roche Diagnostics, Mannheim, Germany) and 10% fetal calf serum (Thermofisher). The samples were sonicated at 20% for 20 s by using a sonifier (Branson, Danbury, CT, USA) and then centrifuged at 16,000 g for 20 min at 4 °C. The supernatant was transferred into new tubes and stored at −80 °C. The abundance of biomarkers was analyzed by using the xMAP technology developed by Luminex (Austin, TX, USA). Their concentrations were determined by using the commercial kits MCYTOMAG-70 K (containing the analytes G-CSF, IFNγ, IL-1α, IL-1β, IL-5, IL-6, IL-10, IL-12 (p40), IL-13, IL-17, KC, and TNFα) and MTH17MAG-47 K (containing the analytes IL-21 and IL-22) from Millipore (Merck KGaA, Darmstadt, Germany) in accordance with the manufacturer’s instructions and subsequently analyzed with five-parameter curve fitting by using the FM3D instrument (Luminex). The final concentrations were determined by inversely predicting (back-fitting) the median fluorescence intensities of the study test samples (as provided by the instrument) by using the five-parameter logistic curve:

$$ f\ \left(x,A,B,C,D,E\right)=D+\frac{A-D}{\ {\left(1+{\left(x/C\right)}^B\right)}^E} $$

The five parameters, *A*, *B*, *C*, *D*, and *E*, were estimated by means of standard calibration curves repeated twice per instrumental run and including seven levels. The test samples were injected into the instrument at two different dilution levels, including two injections (technical replication) per sample and per dilution level. After the appropriate dilution was selected, the two technical replicates were averaged out, and one single quantification per study item (expressed in pg/μL units) was derived for further statistical analysis.

### Statistical evaluation

Body weight was recorded daily during exposure to DSS. In order to study the association of the different exposures with body weight response, we applied a statistical model taking into account intra-animal variation between consecutives days of measures, as well as between group variations. We employed a general additive model with smooth curves adjusted over time by group of exposure, along with fixed effects for exposure groups and random effects for intra-animal variation. A generalized linear model using the Gamma distribution family and log link function was used to test for differences among the DSS groups. For statistical analysis of quantitative non-negative continuous scale data—i.e., DAI and colon weight/length ratio—a generalized linear model using the Gamma distribution family and log link function was used to test for differences among the DSS groups and all groups, respectively. For ordinal data—i.e., diarrhea and colon inflammation scores—a generalized multinomial logit model was used to test for differences among the DSS groups. The results are expressed as mean ± standard error of mean (SEM), and significance was set at *p* < 0.05. For MAP-selected cytokines, differences among the study experimental groups and water were assessed by t-tests and at 5% significance level. No adjustment was made for multiple testing.

## Supplementary information

**Additional file 1: Online Resource 1.** Graphical summary of the study concept and analytical approaches. **Online Resource 2.** Water consumption monitoring (A) water and DSS, (B) nicotine, and (C) anatabine treatment groups. There was no difference in water consumption across the different treatment groups. **Online Resource 3.** Differential gene expression results for pairwise comparisons capturing the pure effects of anatabine/nicotine exposure. The volcano plots (see Fig. 2b) represent the log2 fold changes on the horizontal axis and the corresponding statistical significance –log10 FDR on the vertical axis. The threshold for statistical significance indicated by the colored points is FDR ≤ 0.05. Except for nicotine, the observed signals were rather weak. **Online Resource 4.** Differential gene expression results for various contrasts involving the effects of anatabine/nicotine exposure. Volcano plots for individual gene differential expressions. The volcano plots (see Fig. 2b) represent the log2 fold changes on the horizontal axis and the corresponding statistical significance –log10 FDR on the vertical axis. Similar to the corresponding pure effects of anatabine/nicotine exposure described in Online Resource 2, the observed signals were weak and apparently unaffected by DSS treatment. **Online Resource 5.** Hierarchical representation of the GSA results for all pathways contained in the top Reactome “Immune System” category and for the two-factor “Ana 20*DSS” interaction (larger unannotated version of Fig. 3c). The contents of this plot have been explained in the legends of Fig. 3c and Online Resource 3. Representing all the pathways from the Reactome “Immune System” category enables not only to identify the relevant biological mechanisms, but also to take into account their relationships (i.e. “mechanistic proximity”) at various hierarchical levels, and, complementarily, to examine the parts of the hierarchy that are not involved in the response. The Reactome pathway names corresponding to the node labels are given in the lower part of the figure. **Online Resource 6.** Hierarchical representation of the GSA results for all pathways contained in the top Reactome “Signal Transduction” category and for the two-factor “Ana 20*DSS” interaction. The contents of this plot have been explained in the legends of Fig. 3c and Online Resource 3. The Reactome pathway names corresponding to the node labels are given in the lower part of the figure. **Online Resource 7.** Hierarchical representation of the GSA results for all pathways contained in the top Reactome “Extracellular matrix organization” category and for the two-factor “Ana 20*DSS” interaction. The contents of this plot have been explained in the legends of Fig. 3c and Online Resource 3. The Reactome pathway names corresponding to the node labels are given in the lower part of the figure. **Online Resource 8.** Membership heatmap for all pathways situated downstream of “Signaling by Interleukins” and “Toll−like Receptor Cascades” in the top Reactome “Immune System” category (nodes “3.11” and “3.24” in Fig. 3c and Online Resource 4). For a given pathway p (horizontal axis) and gene g (vertical axis), the values are either 0, 1, or 2. “0” means g does not belong to p; “1” means g belongs to p; and “2” means g is a leading-edge gene of p for the contrast “Ana 20*DSS”. The membership heatmap enables us to explicitly check whether the GSA results (and, in particular, the statistical significance) of pathways situated downstream of a given high-level pathway are driven by the same set of genes and, therefore, do not specifically reflect the hierarchical relationships between the considered pathways. The figure shows that the pathways downstream of “Signaling by Interleukins” are rather independent, whereas the ones downstream of “Toll−like Receptor Cascades” are strongly overlapping and, therefore, reflect the differential expression of the same set of genes. **Online Resource 9.** Membership heatmap for all the pathways situated downstream of “Signaling by GPCR” in the top Reactome “Signal Transduction” category (nodes “2.6” in Online Resource 5). The contents and utility of the membership heatmap have been explained in the legend of Figure S7. The figure shows that the pathways downstream of “Signaling by GPCR” are rather independent, although the fraction of specific genes are quite small in a few cases. **Online Resource 10.** Membership heatmap for all pathways contained in the top “Extracellular matrix organization” category. For a given pathway p (horizontal axis) and gene g (vertical axis), the values are either 0, 1, or 2. “0” means g does not belong to p; “1” means g belongs to p; and “2” means g is a leading-edge gene of p for the contrast “Ana 20*DSS”. The membership heatmap enables us to explicitly check whether the GSA results (and, in particular, the statistical significance) of pathways situated downstream of a given high-level pathway are driven by the same set of genes and, therefore, do not specifically reflect the hierarchical relationships between the considered pathways. The figure shows that the pathways contained in the top “Extracellular matrix organization” category are quite independent. **Online Resource 11.** Heatmap of the leading node perturbations for the TLR–IL1R–TNFR network model. The leading nodes correspond to the network model nodes making the highest contributions to the NPA shown in Fig. 3d. The displayed values correspond to the (positive) percentage NPA contributions (up to a cumulative value of 80%) multiplied by the sign ∈ {− 1, 1} of the node-level perturbations. The annotated rank (starting at 1 for the highest contribution) enables us to identify the most relevant nodes. **Online Resource 12.** Reactome labels for the biological categories “Immune system”, “Metabolism”, “Signal transduction” and “Extracellular matrix organization”.

## Data Availability

The datasets generated and/or analyzed during the current study are available in the ArrayExpress public repository (E-MTAB-8543) and the INTERVALS platform at http://www.intervals.science/studies/, 10.26126/intervals.ixist2.1.

## Abbreviations

ATF: Activating transcription factor
ACTB: β-actin
B2M: β-2 microglobulin
cAMP: Cyclic adenosine monophosphate
CD: Crohn’s disease
cGMP: Cyclic guanosine monophosphate
CREB: CAMP response element-binding protein
DAI: Disease Activity Index
DSS: Dextran sulfate sodium
FDR: False discovery rate
FBLN: Fibulin
FGFR: Fibroblast growth factor receptor
G-CSF: Granulocyte-colony stimulating factor
GAPDH: Glyceraldehyde-3-phosphate dehydrogenase
GPCR: G-protein-coupled receptor
GSA: Gene set analysis
IBD: Inflammatory bowel disease
IL: Interleukin
IL1R: IL-1 receptor
IFN: Interferon
IRAK: IL-1R-associated kinase
JAK: Janus kinase
KC: Keratinocyte chemoattractant
MYD88: Myeloid differentiation primary response 88
LPS: Lipopolysaccharide
MAP: Multi-analyte profiling
MMP: Matrix metalloproteinase
nAChR: Nicotinic acetylcholine receptor
NF: Nuclear factor
NOD1: Nucleotide-binding oligomerization domain-containing protein 1
NPA: Network perturbation amplitude
Nrf: Nuclear factor erythroid 2-related factor
PC: Principal component
PDE: Phosphodiesterase
PI3K: Phosphoinositide 3-kinase
PIK3R: PI3K regulatory subunit
SEM: Standard error of mean
STAT: Signal transducer and activator of transcription
TGF: Transforming growth factor
THBS1: Thrombospondin 1
TIMP: Tissue inhibitor of metalloproteinase
TLR: Toll-like receptor
TNBS: 2,4,6-trinitrobencene sulfonic acid
TNF: Tumor necrosis factor
TNFR: TNF receptor
UC: Ulcerative colitis
ZO: Zonula occludens
