# Supplementary material for: Anatabine ameliorates intestinal inflammation and reduces the production of pro-inflammatory factors in a dextran sulfate sodium mouse model of colitis
Source: J Inflamm (Lond). 2020 Aug 24;17:29. doi: 10.1186/s12950-020-00260-6 (PMC7446176; doi:10.1186/s12950-020-00260-6)
Supplement: Supplementary file 1 — Additional file 1: Online Resource 1. Graphical summary of the study concept and analytical approaches. Online Resource 2. Water consumption monitoring (A) water and DSS, (B) nicotine, and (C) anatabine treatment groups. There was no difference in water consumption across the different treatment groups. Online Resource 3. Differential gene expression results for pairwise comparisons capturing the pure effects of anatabine/nicotine exposure. The volcano plots (see Fig. 2b) represent the log2 fold changes on the horizontal axis and the corresponding statistical significance –log10 FDR on the vertical axis. The threshold for statistical significance indicated by the colored points is FDR ≤ 0.05. Except for nicotine, the observed signals were rather weak. Online Resource 4. Differential gene expression results for various contrasts involving the effects of anatabine/nicotine exposure. Volcano plots for individual gene differential expressions. The volcano plots (see Fig. 2b) represent the log2 fold changes on the horizontal axis and the corresponding statistical significance –log10 FDR on the vertical axis. Similar to the corresponding pure effects of anatabine/nicotine exposure described in Online Resource 2, the observed signals were weak and apparently unaffected by DSS treatment. Online Resource 5. Hierarchical representation of the GSA results for all pathways contained in the top Reactome “Immune System” category and for the two-factor “Ana 20*DSS” interaction (larger unannotated version of Fig. 3c). The contents of this plot have been explained in the legends of Fig. 3c and Online Resource 3. Representing all the pathways from the Reactome “Immune System” category enables not only to identify the relevant biological mechanisms, but also to take into account their relationships (i.e. “mechanistic proximity”) at various hierarchical levels, and, complementarily, to examine the parts of the hierarchy that are not involved in the response. The Reactome pathway names [file 12950_2020_260_MOESM1_ESM.pdf]

Online Resource 1

Graphical summary of the study concept and analytical approaches

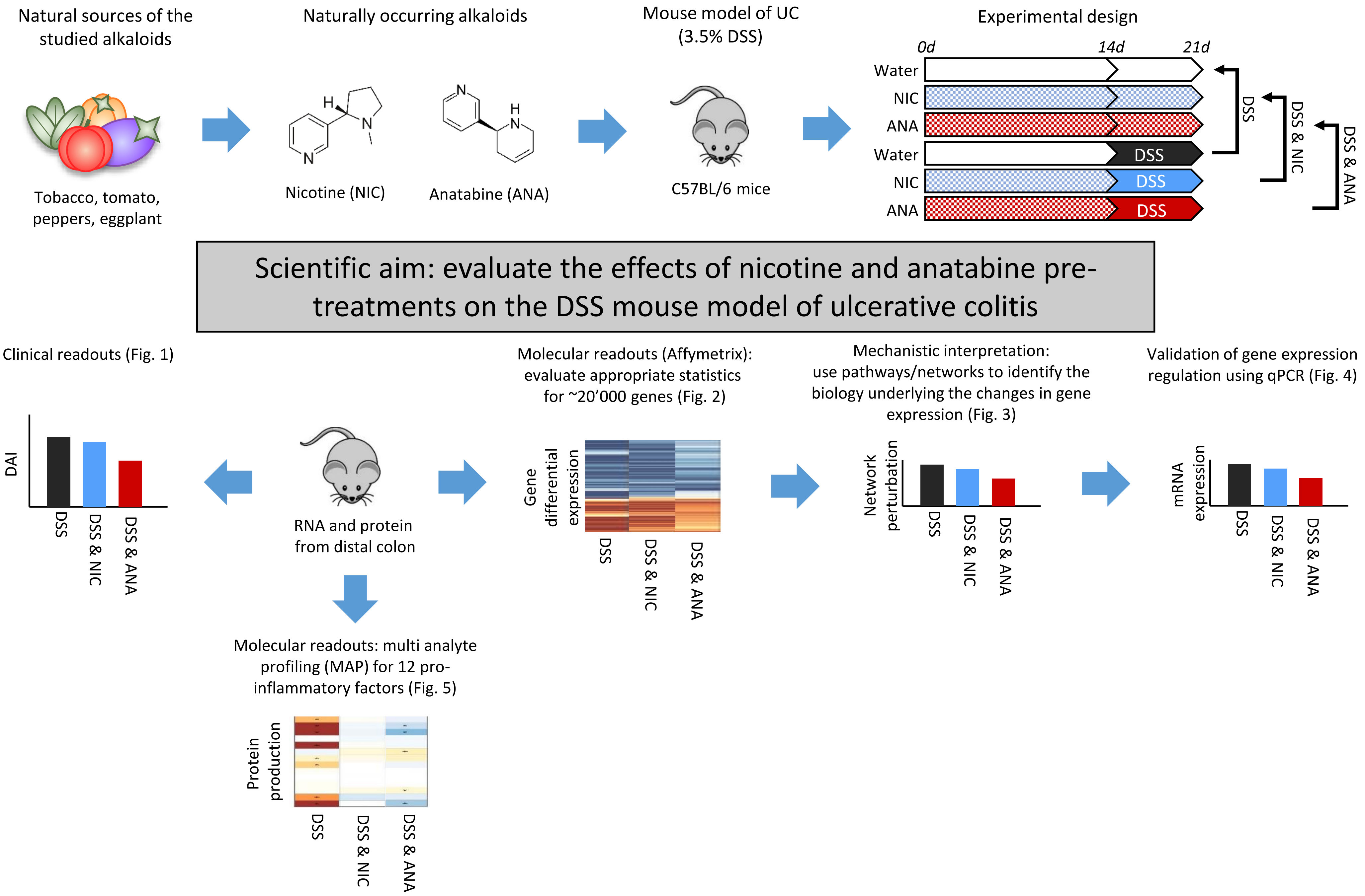

Online Resource 2

Water consumption monitoring (A) water and DSS, (B) nicotine, and (C) anatabine treatment groups. There was no difference in water consumption across the different treatment groups.

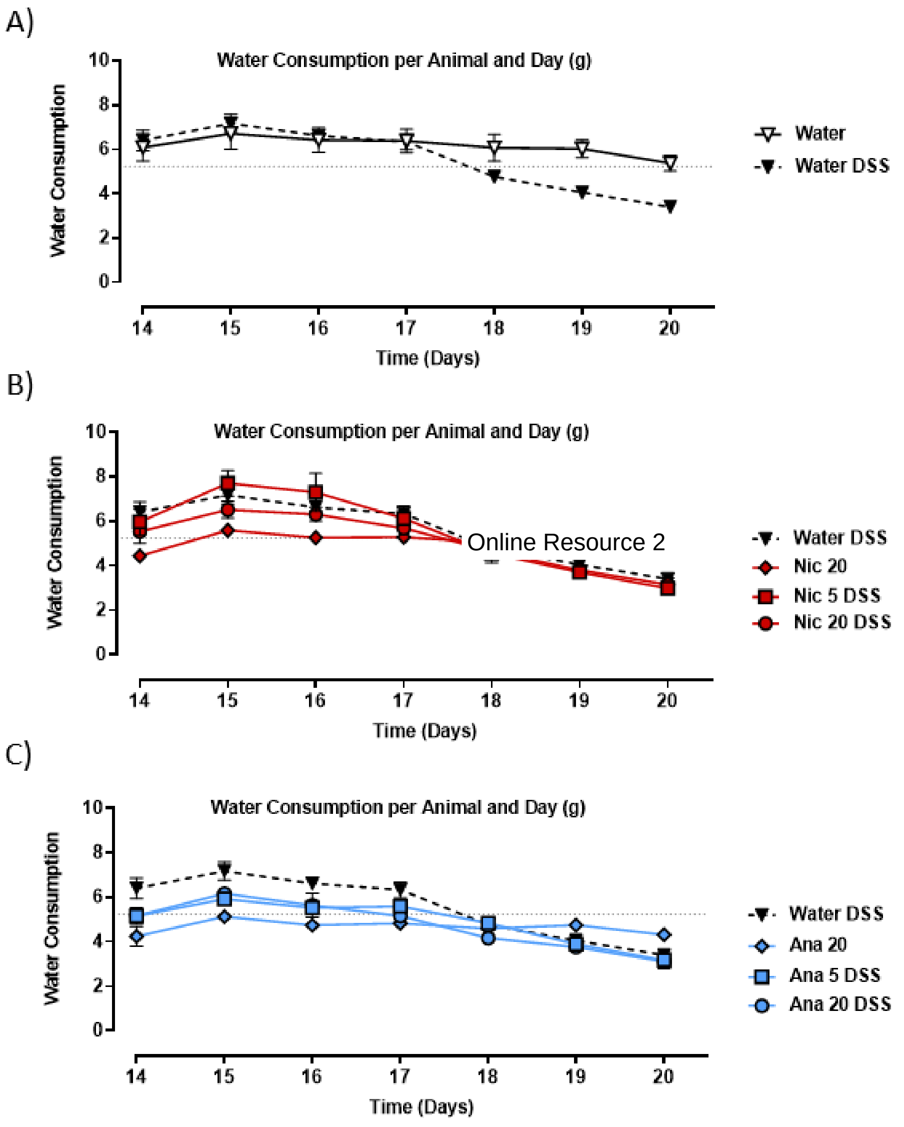

Online Resource 3

Differential gene expression results for pairwise comparisons capturing the pure effects of anatabine/nicotine exposure. The volcano plots (see Fig. 2B) represent the log2 fold changes on the horizontal axis and the corresponding statistical significance –log10 FDR on the vertical axis. The threshold for statistical significance indicated by the colored points is FDR ≤ 0.05. Except for nicotine, the observed signals were rather weak.

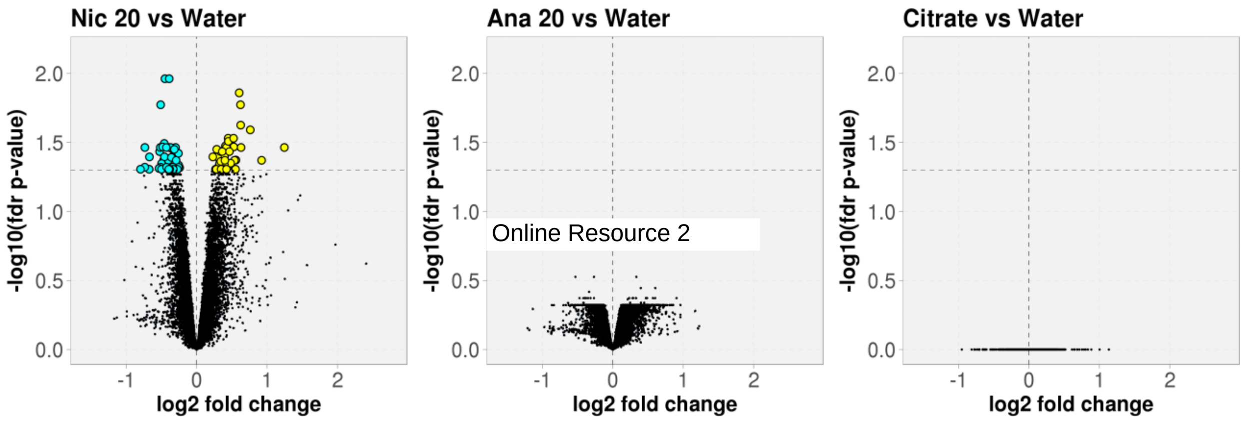

Online Resource 4

Differential gene expression results for various contrasts involving the effects of anatabine/nicotine exposure. Volcano plots for individual gene differential expressions. The volcano plots (see Fig. 2B) represent the log2 fold changes on the horizontal axis and the corresponding statistical significance –log10 FDR on the vertical axis. Similar to the corresponding pure effects of anatabine/nicotine exposure described in Online Resource 2, the observed signals were weak and apparently unaffected by DSS treatment.

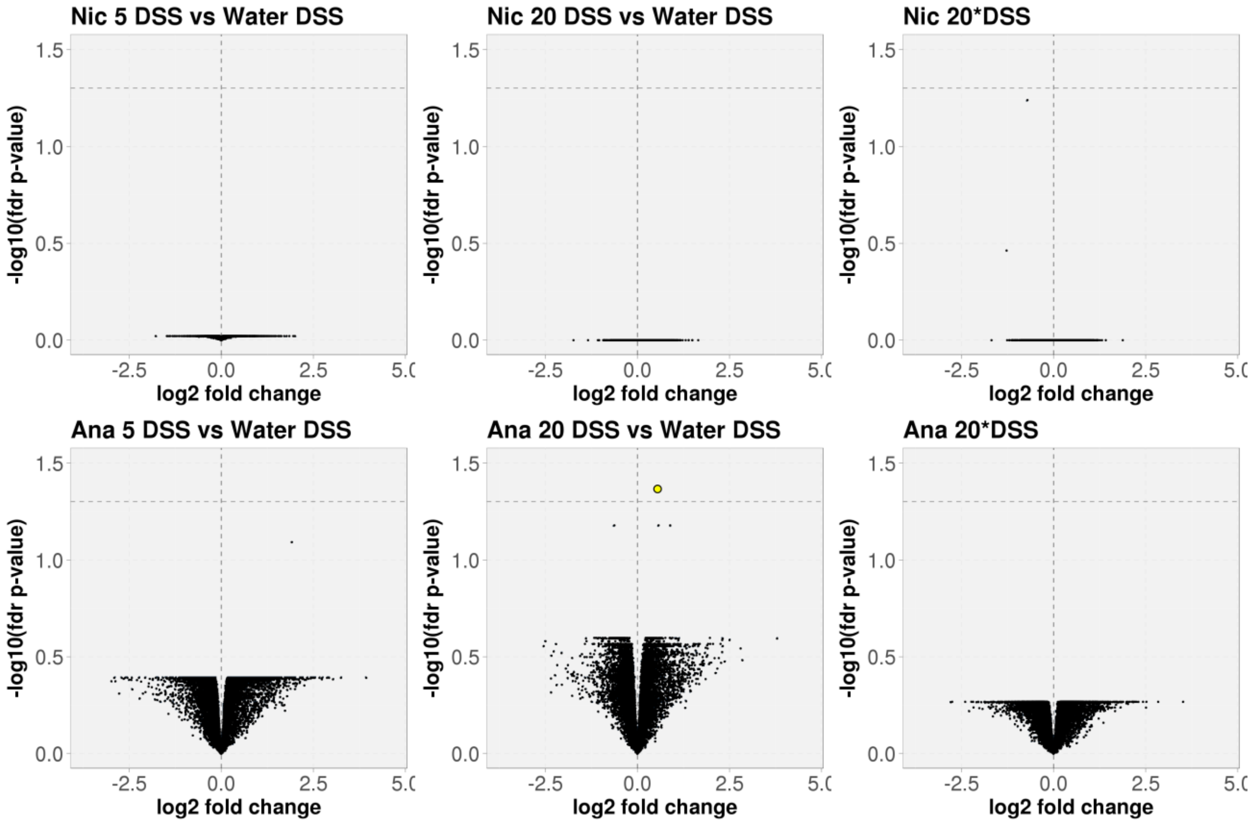

Online Resource 5

Hierarchical representation of the GSA results for all pathways contained in the top Reactome “Immune System” category and for the two-factor “Ana 20*DSS” interaction (larger unannotated version of Fig. 3C). The contents of this plot have been explained in the legends of Fig. 3C and Online Resource 3. Representing all the pathways from the Reactome “Immune System” category enables not only to identify the relevant biological mechanisms, but also to take into account their relationships (i.e. “mechanistic proximity”) at various hierarchical levels, and, complementarily, to examine the parts of the hierarchy that are not involved in the response. The Reactome pathway names corresponding to the node labels are given in the lower part of the figure.

Immune System

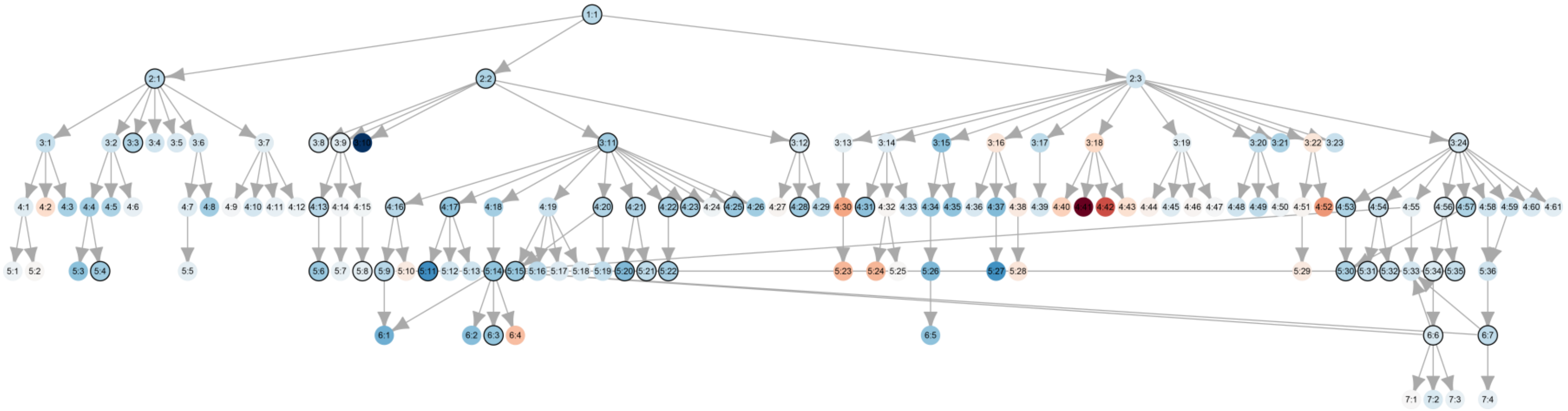

Online Resource 5 continued

For more details please refer to Online Resource 12.

1:1	Immune System	3:4	MHC class II antigen presentation
2:1	Adaptive Immune System	3:5	Rap1 signalling
2:2	Cytokine Signaling in Immune system	3:6	Signaling by the B Cell Receptor (BCR)
2:3	Innate Immune System	3:7	TCR signaling
3:1	Class I MHC mediated antigen processing & presentation	3:8	Growth hormone receptor signaling
3:2	Costimulation by the CD28 family	3:9	Interferon Signaling
3:3	Immunoregulatory interactions between a Lymphoid and a non-Lymphoid cell	3:10	Prolactin receptor signaling

3:11	Signaling by Interleukins	3:18	DDX58/IFIH1-mediated induction of interferon-alpha/beta
3:12	TNFR2 non-canonical NF-kB pathway	3:19	Fc epsilon receptor (FCERI) signaling
3:13	Antimicrobial peptides	3:20	Fcgamma receptor (FCGR) dependent phagocytosis
3:14	C-type lectin receptors (CLRs)	3:21	Neutrophil degranulation
3:15	Complement cascade	3:22	Nucleotide-binding domain, leucine rich repeat containing receptor (NLR) signaling pathways
3:16	Cytosolic sensors of pathogen-associated DNA	3:23	ROS and RNS production in phagocytes
3:17	DAP12 interactions	3:24	Toll-like Receptor Cascades

Online Resource 6

Hierarchical representation of the GSA results for all pathways contained in the top Reactome “Signal Transduction” category and for the two-factor “Ana 20*DSS” interaction. The contents of this plot have been explained in the legends of Fig. 3C and Online Resource 3. The Reactome pathway names corresponding to the node labels are given in the lower part of the figure.

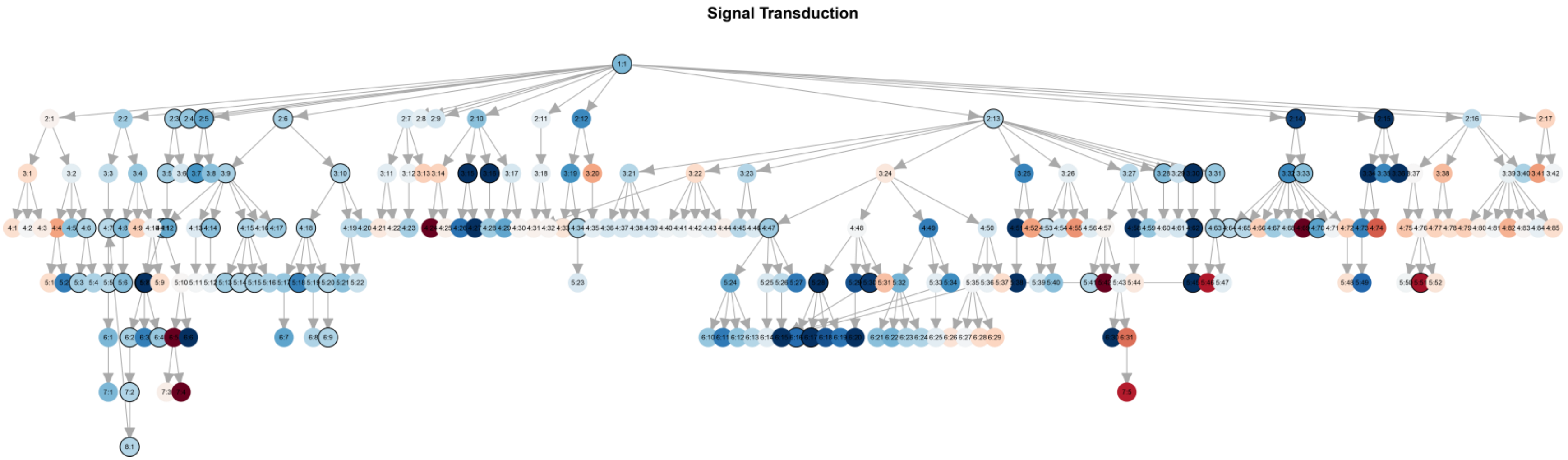

Online Resource 6 continued

For more details see Online Resource 12.

1:1	Signal Transduction	3:1	TNF signaling
2:1	Death Receptor Signalling	3:2	p75 NTR receptor-mediated signalling
2:2	Intracellular signaling by second messengers	3:3	DAG and IP3 signaling
2:3	MAPK family signaling cascades	3:4	PIP3 activates AKT signaling
2:4	Integrin signaling	3:5	MAPK1/MAPK3 signaling
2:5	Signaling by Erythropoietin	3:6	MAPK6/MAPK4 signaling
2:6	Signaling by GPCR	3:7	Erythropoietin activates Phosphoinositide-3-kinase (PI3K)
2:7	Signaling by Hedgehog	3:8	Erythropoietin activates RAS
2:8	Signaling by Hippo	3:9	GPCR downstream signalling
2:9	Signaling by Leptin	3:10	GPCR ligand binding
2:10	Signaling by NOTCH	3:11	Hedgehog 'off' state
2:11	Signaling by Non-Receptor Tyrosine Kinases	3:12	Hedgehog 'on' state
2:12	Signaling by Nuclear Receptors	3:13	Hedgehog ligand biogenesis
2:13	Signaling by Receptor Tyrosine Kinases	3:14	Pre-NOTCH Expression and Processing
2:14	Signaling by Rho GTPases	3:15	Signaling by NOTCH1
2:15	Signaling by TGF-beta family members	3:16	Signaling by NOTCH3
2:16	Signaling by WNT	3:17	Signaling by NOTCH4
2:17	mTOR signalling	3:18	Signaling by PTK6
		3:19	ESR-mediated signaling

Online Resource 7

Hierarchical representation of the GSA results for all pathways contained in the top Reactome “Extracellular matrix organization” category and for the two-factor “Ana 20*DSS” interaction. The contents of this plot have been explained in the legends of Fig. 3C and Online Resource 3. The Reactome pathway names corresponding to the node labels are given in the lower part of the figure.

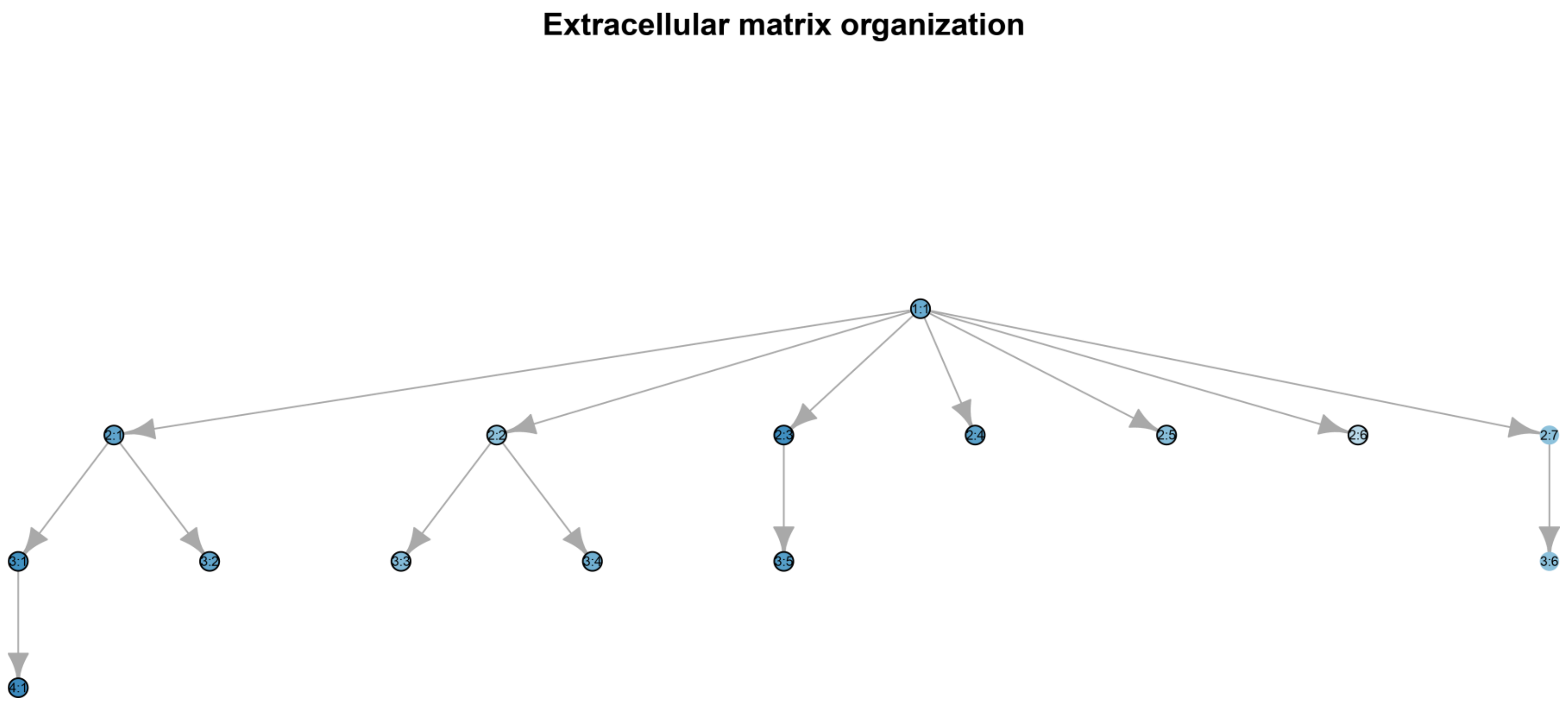

Online Resource 7 continued

For more details see Online Resource 12.

3:1	Collagen biosynthesis and modifying enzymes
4:1	Collagen chain trimerization
2:1	Collagen formation
3:2	Assembly of collagen fibrils and other multimeric structures
2:2	Degradation of the extracellular matrix
3:3	Activation of Matrix Metalloproteinases
3:4	Collagen degradation
2:3	Elastic fibre formation
3:5	Molecules associated with elastic fibres
1:1	Extracellular matrix organization
2:4	ECM proteoglycans
2:5	Integrin cell surface interactions
2:6	Laminin interactions
2:7	Non-integrin membrane-ECM interactions
3:6	Syndecan interactions

Online Resource 8

Membership heatmap for all pathways situated downstream of “Signaling by Interleukins” and “Toll−like Receptor Cascades” in the top Reactome “Immune System” category (nodes “3.11” and “3.24” in Fig. 3C and Online Resource 4). For a given pathway p (horizontal axis) and gene g (vertical axis), the values are either 0, 1, or 2. “0” means g does not belong to p; “1” means g belongs to p; and “2” means g is a leading-edge gene of p for the contrast “Ana 20*DSS”. The membership heatmap enables us to explicitly check whether the GSA results (and, in particular, the statistical significance) of pathways situated downstream of a given high-level pathway are driven by the same set of genes and, therefore, do not specifically reflect the hierarchical relationships between the considered pathways. The figure shows that the pathways downstream of “Signaling by Interleukins” are rather independent, whereas the ones downstream of “Toll−like Receptor Cascades” are strongly overlapping and, therefore, reflect the differential expression of the same set of genes.

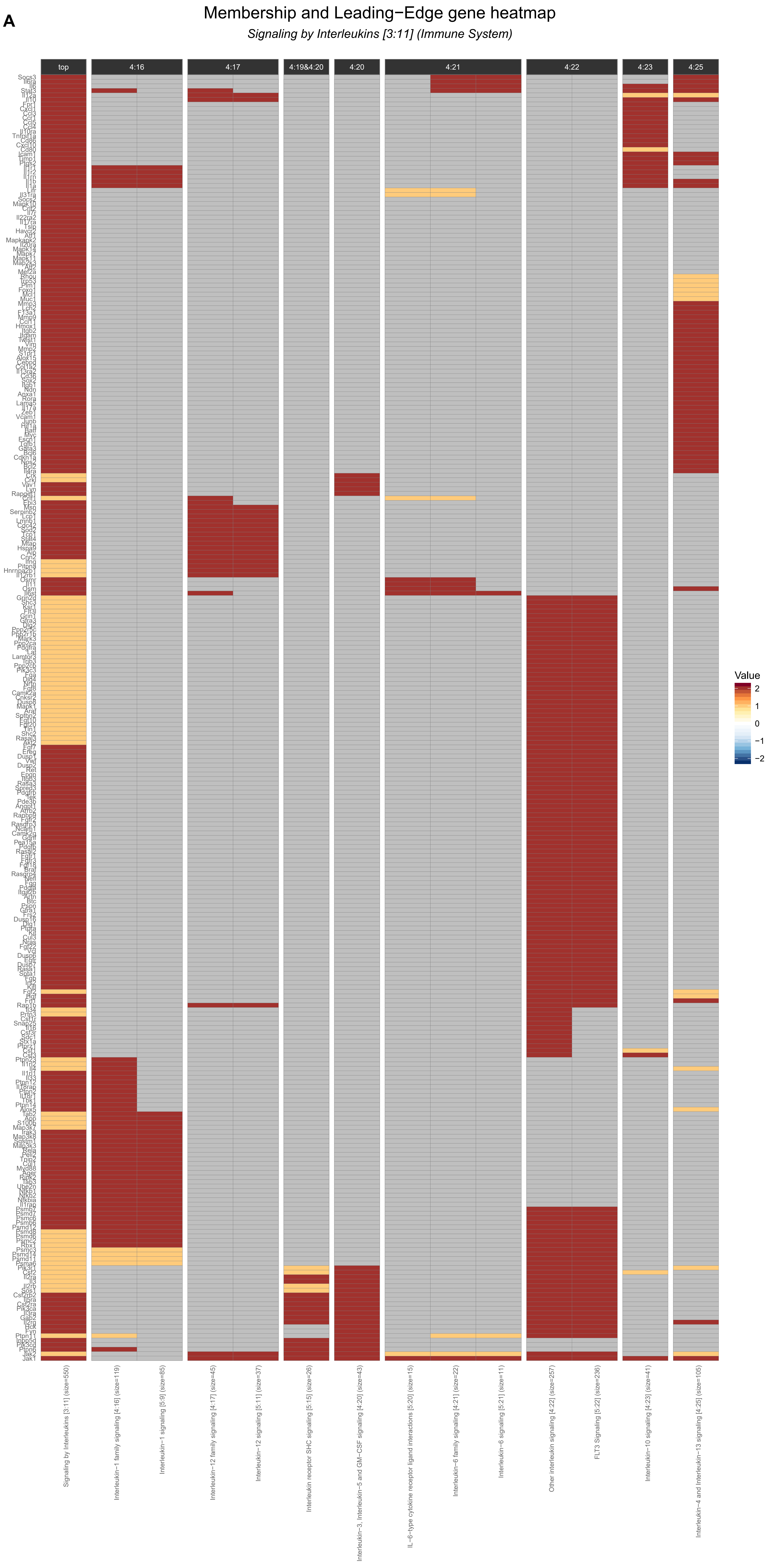

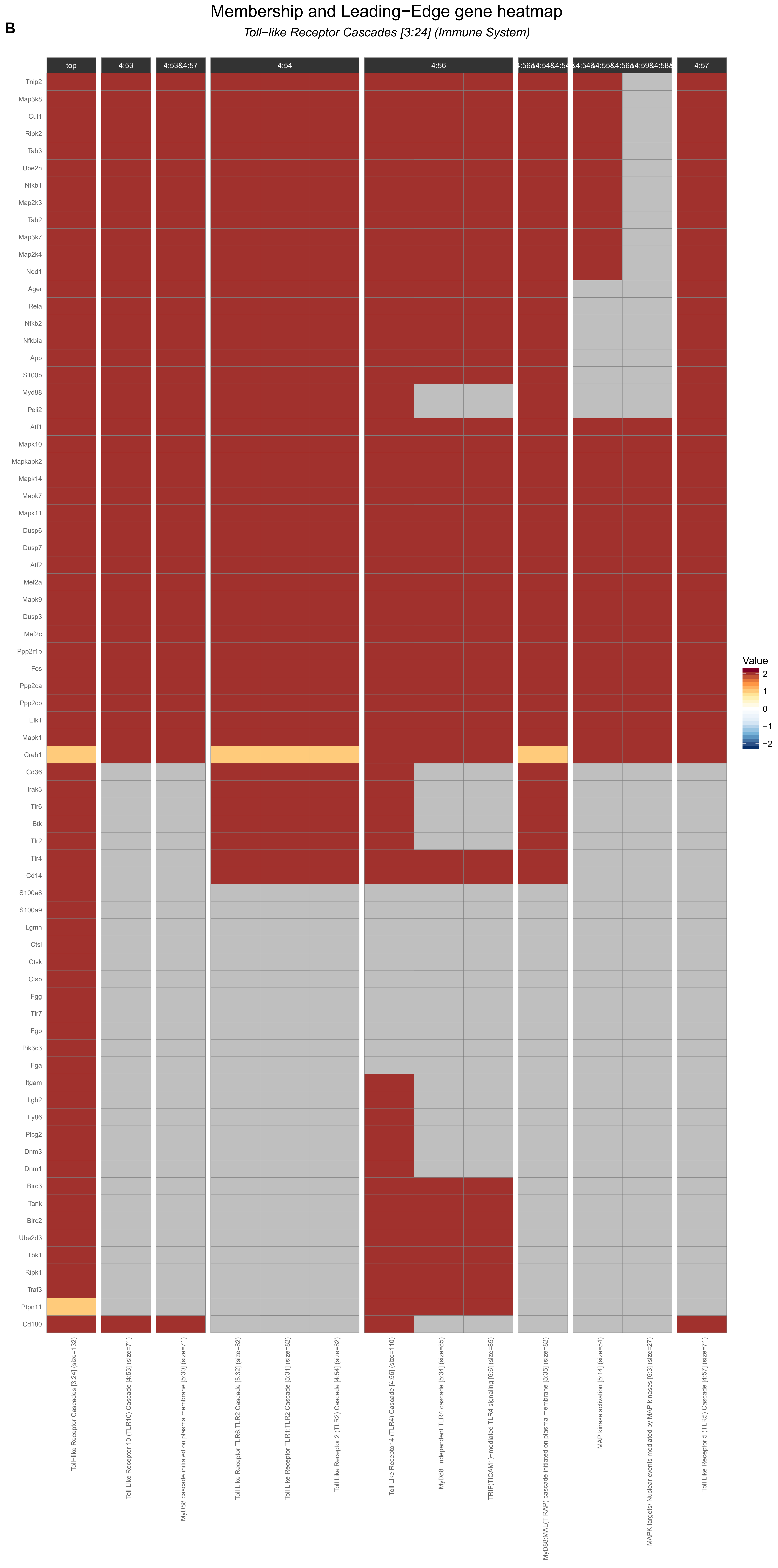
B
Membership and Leading−Edge gene heatmap
Toll−like Receptor Cascades [3:24] (Immune System)
top
4:53
4:53&4:57
4:54
4:56
4:57
Tnip2
Map3k8
Cul1
Ripk2
Tab3
Ube2n
Nfkb1
Map2k3
Tab2
Map3k7
Map2k4
Nod1
Ager
Rela
Nfkb2
Nfkbia
App
S100b
Myd88
Peli2
Atf1
Mapk10
Mapkapk2
Mapk14
Mapk7
Mapk11
Dusp6
Dusp7
Atf2
Mef2a
Mapk9
Dusp3
Mef2c
Ppp2r1b
Fos
Ppp2ca
Ppp2cb
Elk1
Mapk1
Creb1
Cd36
Irak3
Tlr6
Btk
Tlr2
Tlr4
Cd14
S100a8
S100a9
Lgmn
Ctsl
Ctsk
Ctsb
Fgg
Tlr7
Fgb
Pik3c3
Fga
Itgam
Itgb2
Ly86
Plcg2
Dnm3
Dnm1
Birc3
Tank
Birc2
Ube2d3
Tbk1
Ripk1
Traf3
Ptpn11
Cd180
Toll−like Receptor Cascades [3:24] (size=132)
Toll Like Receptor 10 (TLR10) Cascade [4:53] (size=71)
MyD88 cascade initiated on plasma membrane [5:30] (size=71)
Toll Like Receptor TLR6:TLR2 Cascade [5:32] (size=82)
Toll Like Receptor TLR1:TLR2 Cascade [5:31] (size=82)
Toll Like Receptor 2 (TLR2) Cascade [4:54] (size=82)
Toll Like Receptor 4 (TLR4) Cascade [4:56] (size=110)
MyD88−independent TLR4 cascade [5:34] (size=85)
TRIF(TICAM1)−mediated TLR4 signaling [6:6] (size=85)
MyD88:MAL(TIRAP) cascade initiated on plasma membrane [5:35] (size=82)
MAP kinase activation [5:14] (size=54)
MAPK targets/ Nuclear events mediated by MAP kinases [6:3] (size=27)
Toll Like Receptor 5 (TLR5) Cascade [4:57] (size=71)
Value
2
1
0
−1
−2

Online Resource 9

Membership heatmap for all the pathways situated downstream of “Signaling by GPCR” in the top Reactome “Signal Transduction” category (nodes “2.6” in Online Resource 5). The contents and utility of the membership heatmap have been explained in the legend of Figure S7. The figure shows that the pathways downstream of “Signaling by GPCR” are rather independent, although the fraction of specific genes are quite small in a few cases.

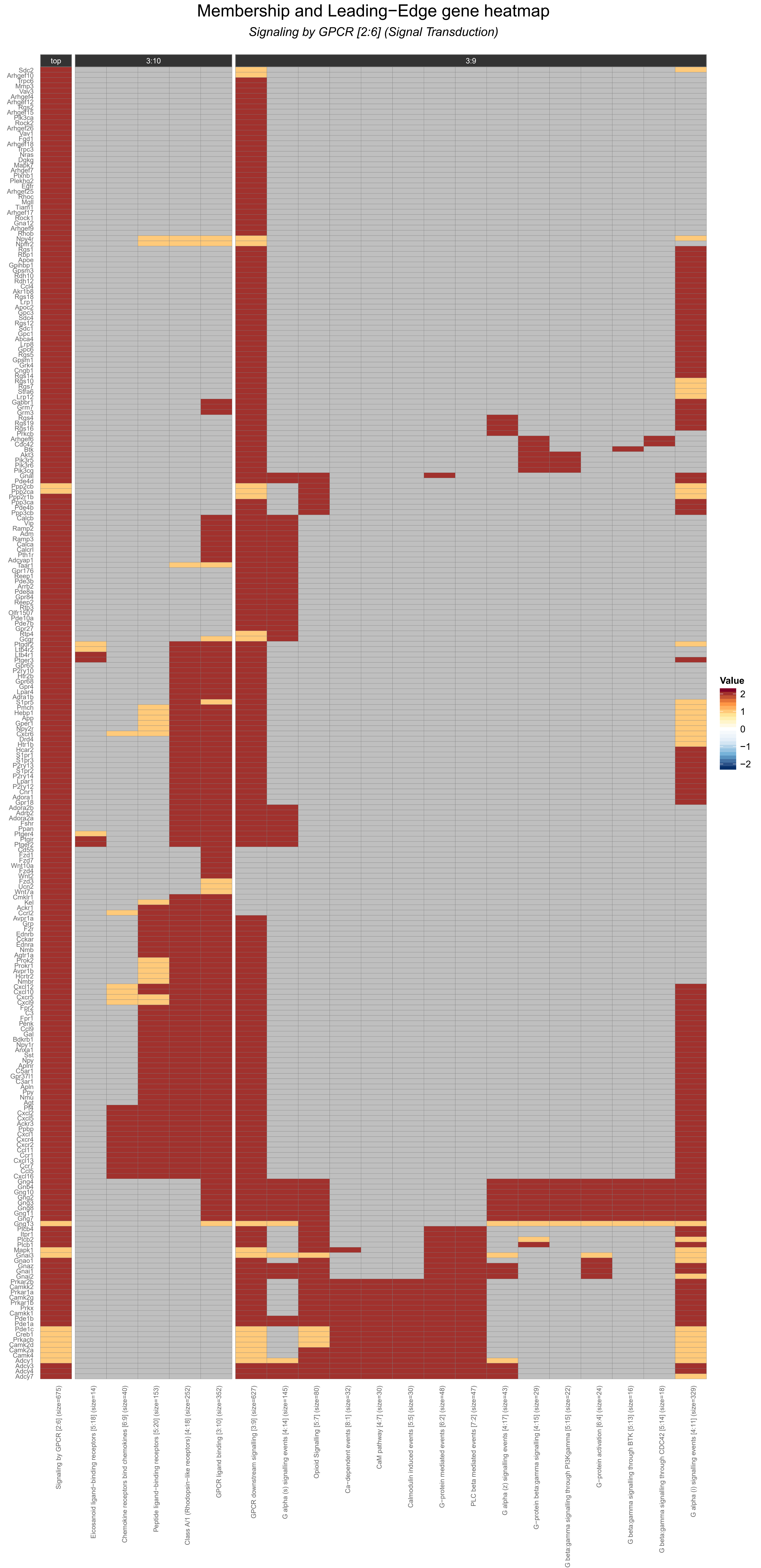

Online Resource 10

Membership heatmap for all pathways contained in the top “Extracellular matrix organization” category. For a given pathway p (horizontal axis) and gene g (vertical axis), the values are either 0, 1, or 2. “0” means g does not belong to p; “1” means g belongs to p; and “2” means g is a leading-edge gene of p for the contrast “Ana 20*DSS”. The membership heatmap enables us to explicitly check whether the GSA results (and, in particular, the statistical significance) of pathways situated downstream of a given high-level pathway are driven by the same set of genes and, therefore, do not specifically reflect the hierarchical relationships between the considered pathways. The figure shows that the pathways contained in the top “Extracellular matrix organization” category are quite independent.

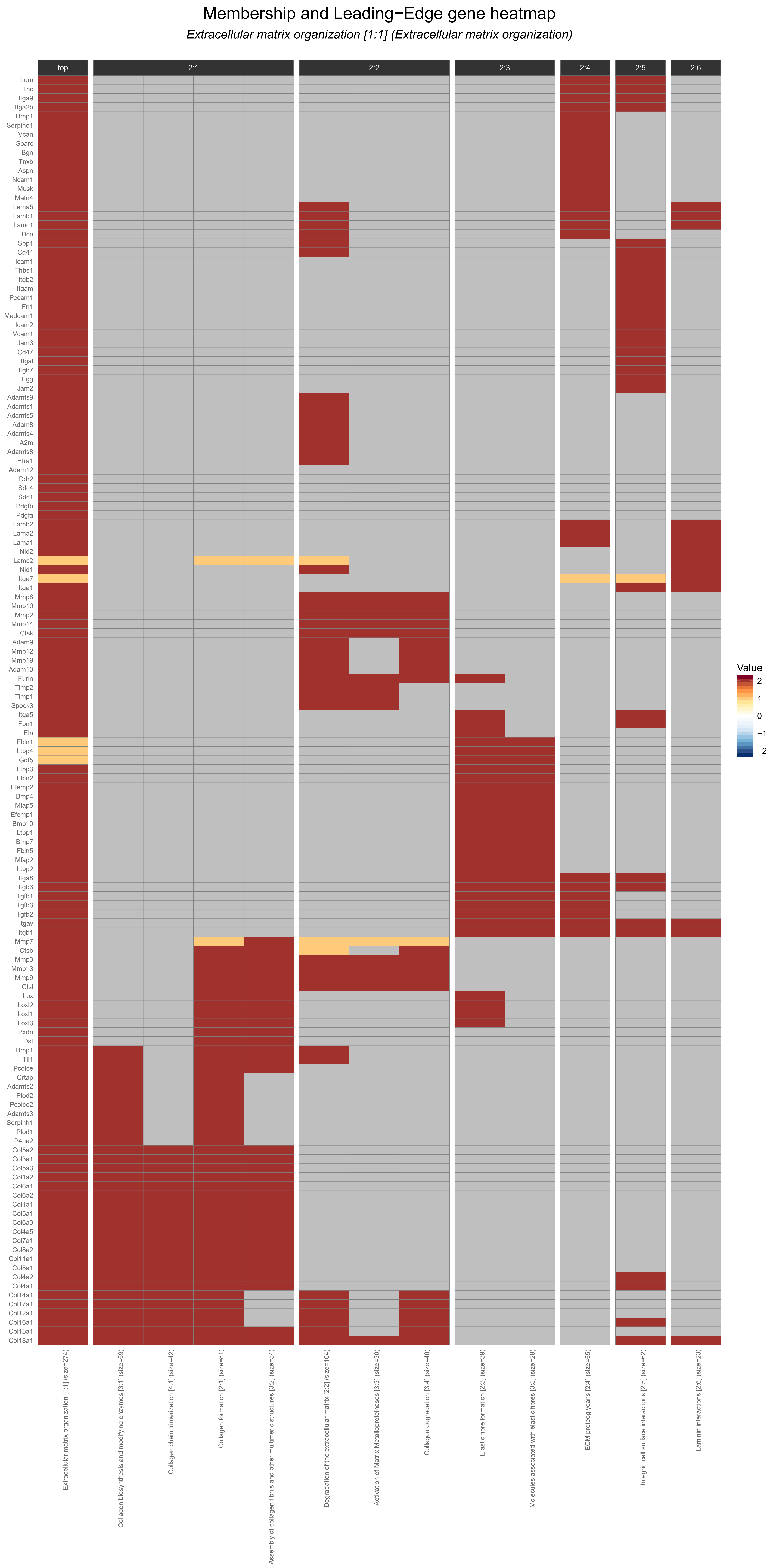

Online Resource 11

Heatmap of the leading node perturbations for the TLR–IL1R–TNFR network model. The leading nodes correspond to the network model nodes making the highest contributions to the NPA shown in Fig. 3D. The displayed values correspond to the (positive) percentage NPA contributions (up to a cumulative value of 80%) multiplied by the sign ∈ {-1, 1} of the node-level perturbations. The annotated rank (starting at 1 for the highest contribution) enables us to identify the most relevant nodes.

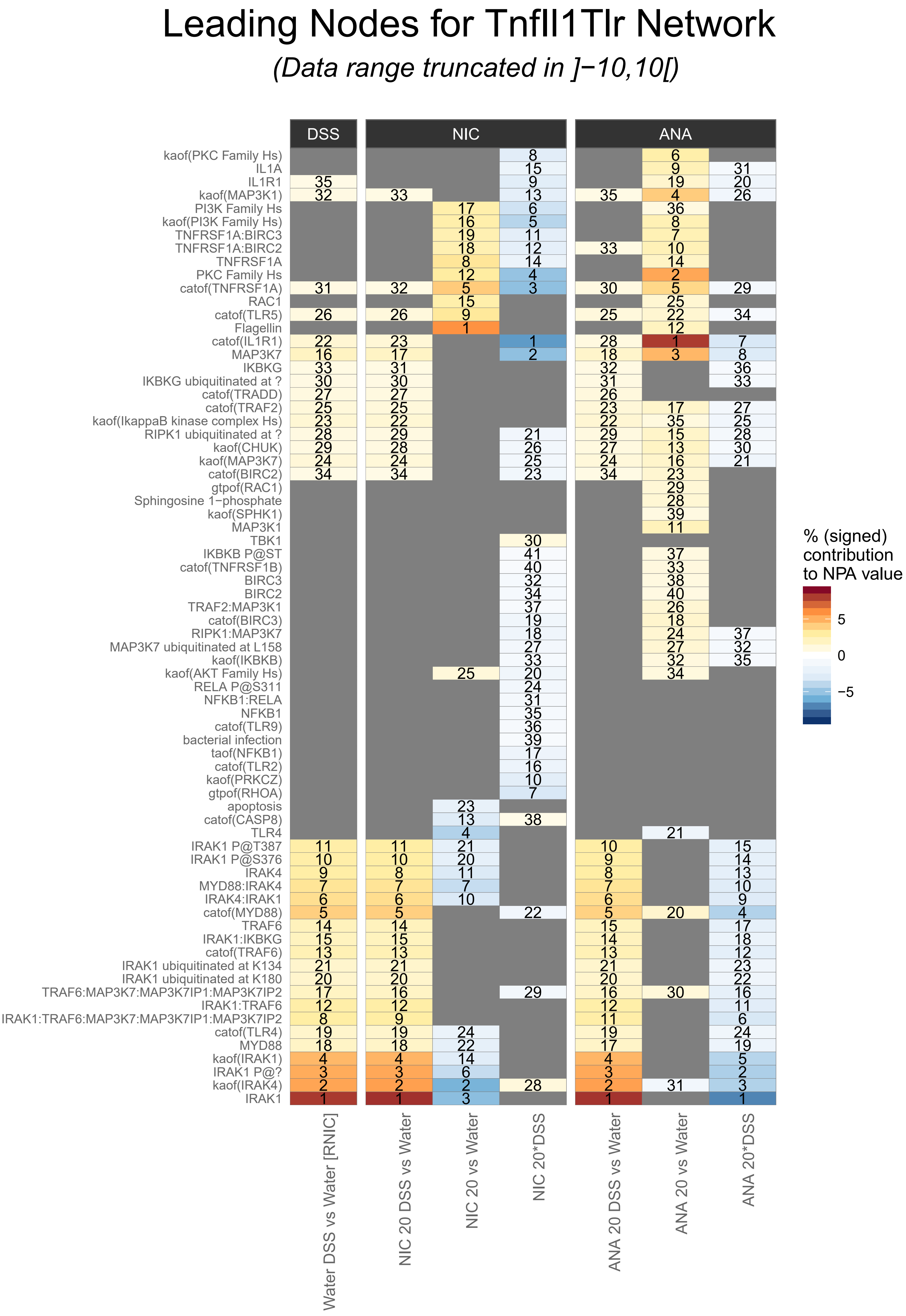

Online Resource 12

Reactome labels

**Immune System**	x
Adaptive Immune System	2:1
Class I MHC mediated antigen processing & presentation	3:1
Costimulation by the CD28 family	3:2
Immunoregulatory interactions between a Lymphoid and a non-Lymphoid cell	3:3
MHC class II antigen presentation	3:4
Rap1 signalling	3:5
Signaling by the B Cell Receptor (BCR)	3:6
TCR signaling	3:7
Antigen processing-Cross presentation	4:1
Cross-presentation of soluble exogenous antigens (endosomes)	5:1
ER-Phagosome pathway	5:2
Antimicrobial peptides	3:13
Defensins	4:30
Antiviral mechanism by IFN-stimulated genes	4:13
ISG15 antiviral mechanism	5:6
C-type lectin receptors (CLRs)	3:14
CD209 (DC-SIGN) signaling	4:31
CLEC7A (Dectin-1) signaling	4:32
Dectin-2 family	4:33
CD28 co-stimulation	4:4
CD28 dependent PI3K/Akt signaling	5:3
CD28 dependent Vav1 pathway	5:4
CLEC7A (Dectin-1) induces NFAT activation	5:24
Dectin-1 mediated noncanonical NF-kB signaling	5:25
Antigen Presentation: Folding, assembly and peptide loading of class I MHC	4:2
Antigen processing: Ubiquitination & Proteasome degradation	4:3
Complement cascade	3:15
Initial triggering of complement	4:34
Regulation of Complement cascade	4:35
CTLA4 inhibitory signaling	4:5
PD-1 signaling	4:6
Creation of C4 and C2 activators	5:26
Classical antibody-mediated complement activation	6:5
Cytokine Signaling in Immune system	2:2
Growth hormone receptor signaling	3:8
Interferon Signaling	3:9
Prolactin receptor signaling	3:10
Signaling by Interleukins	3:11
TNFR2 non-canonical NF-kB pathway	3:12
Cytosolic sensors of pathogen-associated DNA	3:16
Regulation of innate immune responses to cytosolic DNA	4:36
STING mediated induction of host immune responses	4:37
ZBP1(DAI) mediated induction of type I IFNs	4:38
DAP12 interactions	3:17
DAP12 signaling	4:39

DDX58/IFIH1-mediated induction of interferon-alpha/beta	3:18
Negative regulators of DDX58/IFIH1 signaling	4:40
TRAF3-dependent IRF activation pathway	4:41
TRAF6 mediated IRF7 activation	4:42
TRAF6 mediated NF-kB activation	4:43
Beta defensins	5:23
Downstream signaling events of B Cell Receptor (BCR)	4:7
Activation of NF-kappaB in B cells	5:5
Fc epsilon receptor (FCERI) signaling	3:19
FCERI mediated Ca+2 mobilization	4:44
FCERI mediated MAPK activation	4:45
FCERI mediated NF-kB activation	4:46
Role of LAT2/NTAL/LAB on calcium mobilization	4:47
Fcgamma receptor (FCGR) dependent phagocytosis	3:20
FCGR activation	4:48
Regulation of actin dynamics for phagocytic cup formation	4:49
Role of phospholipids in phagocytosis	4:50
Immune System	1:1
Innate Immune System	2:3
Inflammasomes	4:51
The NLRP3 inflammasome	5:29
Neutrophil degranulation	3:21
Nucleotide-binding domain, leucine rich repeat containing receptor (NLR) signaling pathways	3:22
ROS and RNS production in phagocytes	3:23
Toll-like Receptor Cascades	3:24
Interferon alpha/beta signaling	4:14
Interferon gamma signaling	4:15
Regulation of IFNA signaling	5:7
Regulation of IFNG signaling	5:8
Interleukin-1 family signaling	4:16
Interleukin-1 signaling	5:9
Interleukin-37 signaling	5:10
MAP3K8 (TPL2)-dependent MAPK1/3 activation	6:1
TAK1 activates NFkB by phosphorylation and activation of IKKs complex	5:33
Interleukin-12 family signaling	4:17
Interleukin-12 signaling	5:11
Interleukin-27 signaling	5:12
Interleukin-35 Signalling	5:13
Interleukin-17 signaling	4:18
MAP kinase activation	5:14
Interleukin-2 family signaling	4:19
Interleukin receptor SHC signaling	5:15
Interleukin-15 signaling	5:16
Interleukin-2 signaling	5:17
Interleukin-21 signaling	5:18
Interleukin-3, Interleukin-5 and GM-CSF signaling	4:20
Regulation of signaling by CBL	5:19
Interleukin-6 family signaling	4:21
IL-6-type cytokine receptor ligand interactions	5:20

Interleukin-6 signaling	5:21
JNK (c-Jun kinases) phosphorylation and activation mediated by activated human TAK1	6:2
MAPK targets/ Nuclear events mediated by MAP kinases	6:3
activated TAK1 mediates p38 MAPK activation	6:4
MyD88 cascade initiated on plasma membrane	5:30
MyD88 dependent cascade initiated on endosome	5:36
TRAF6 mediated induction of NFkB and MAP kinases upon TLR7/8 or 9 activation	6:7
MyD88-independent TLR4 cascade	5:34
TRIF(TICAM1)-mediated TLR4 signaling	6:6
MyD88:MAL(TIRAP) cascade initiated on plasma membrane	5:35
NOD1/2 Signaling Pathway	4:52
Other interleukin signaling	4:22
FLT3 Signaling	5:22
IRF3-mediated induction of type I IFN	5:27
Interleukin-10 signaling	4:23
Interleukin-20 family signaling	4:24
Interleukin-4 and Interleukin-13 signaling	4:25
Interleukin-7 signaling	4:26
Antigen activates B Cell Receptor (BCR) leading to generation of second messengers	4:8
Downstream TCR signaling	4:9
Generation of second messenger molecules	4:10
Phosphorylation of CD3 and TCR zeta chains	4:11
Translocation of ZAP-70 to Immunological synapse	4:12
NIK-->noncanonical NF-kB signaling	4:27
TNF receptor superfamily (TNFSF) members mediating non-canonical NF-kB pathway	4:28
TNFs bind their physiological receptors	4:29
IRAK2 mediated activation of TAK1 complex upon TLR7/8 or 9 stimulation	7:4
Activation of IRF3/IRF7 mediated by TBK1/IKK epsilon	7:1
IKK complex recruitment mediated by RIP1	7:2
TRAF6-mediated induction of TAK1 complex within TLR4 complex	7:3
Toll Like Receptor 10 (TLR10) Cascade	4:53
Toll Like Receptor 2 (TLR2) Cascade	4:54
Toll Like Receptor TLR1:TLR2 Cascade	5:31
Toll Like Receptor TLR6:TLR2 Cascade	5:32
Toll Like Receptor 3 (TLR3) Cascade	4:55
Toll Like Receptor 4 (TLR4) Cascade	4:56
Toll Like Receptor 5 (TLR5) Cascade	4:57
Toll Like Receptor 7/8 (TLR7/8) Cascade	4:58
Toll Like Receptor 9 (TLR9) Cascade	4:59
Regulation of TLR by endogenous ligand	4:60
Trafficking and processing of endosomal TLR	4:61
RIP-mediated NFkB activation via ZBP1	5:28

**Extracellular matrix organization**	x
Collagen biosynthesis and modifying enzymes	3:1
Collagen chain trimerization	4:1
Collagen formation	2:1
Assembly of collagen fibrils and other multimeric structures	3:2
Degradation of the extracellular matrix	2:2
Activation of Matrix Metalloproteinases	3:3
Collagen degradation	3:4
Elastic fibre formation	2:3
Molecules associated with elastic fibres	3:5
Extracellular matrix organization	1:1
ECM proteoglycans	2:4
Integrin cell surface interactions	2:5
Laminin interactions	2:6
Non-integrin membrane-ECM interactions	2:7
Syndecan interactions	3:6

**Signal Transduction**	x
Amine ligand-binding receptors	5:17
Serotonin receptors	6:7
Beta-catenin independent WNT signaling	3:37
Ca2+ pathway	4:75
PCP/CE pathway	4:76
Ca-dependent events	8:1
CaM pathway	4:7
Calmodulin induced events	5:5
PKA-mediated phosphorylation of CREB	6:1
Cell death signalling via NRAGE, NRIF and NADE	4:4
NRAGE signals death through JNK	5:1
NRIF signals cell death from the nucleus	5:2
Class A/1 (Rhodopsin-like receptors)	4:18
Eicosanoid ligand-binding receptors	5:18
Nucleotide-like (purinergic) receptors	5:19
Peptide ligand-binding receptors	5:20
Class B/2 (Secretin family receptors)	4:19
Calcitonin-like ligand receptors	5:21
Glucagon-type ligand receptors	5:22
DAG and IP3 signaling	3:3
Death Receptor Signalling	2:1
TNF signaling	3:1
p75 NTR receptor-mediated signalling	3:2
Degradation of beta-catenin by the destruction complex	3:38
Beta-catenin phosphorylation cascade	4:77
Repression of WNT target genes	4:78
Downstream signaling of activated FGFR1	5:24
FRS-mediated FGFR1 signaling	6:10
PI-3K cascade:FGFR1	6:11
Phospholipase C-mediated cascade: FGFR1	6:12
SHC-mediated cascade:FGFR1	6:13
Downstream signaling of activated FGFR2	5:28
FRS-mediated FGFR2 signaling	6:16
PI-3K cascade:FGFR2	6:17
Phospholipase C-mediated cascade; FGFR2	6:18
SHC-mediated cascade:FGFR2	6:19
Downstream signaling of activated FGFR3	5:32
FRS-mediated FGFR3 signaling	6:21
PI-3K cascade:FGFR3	6:22
Phospholipase C-mediated cascade; FGFR3	6:23
SHC-mediated cascade:FGFR3	6:24
Downstream signaling of activated FGFR4	5:35
FRS-mediated FGFR4 signaling	6:26
PI-3K cascade:FGFR4	6:27
Phospholipase C-mediated cascade; FGFR4	6:28
SHC-mediated cascade:FGFR4	6:29
ESR-mediated signaling	3:19
Estrogen-dependent gene expression	4:33
Extra-nuclear estrogen signaling	4:34
Estrogen-dependent nuclear events downstream of ESR-membrane signaling	5:23

FGFR1 ligand binding and activation	5:25
FGFR1c ligand binding and activation	6:14
FGFR2 ligand binding and activation	5:29
FGFR2c ligand binding and activation	6:20
FGFR3 ligand binding and activation	5:33
FGFR3c ligand binding and activation	6:25
G alpha (i) signalling events	4:11
Opioid Signalling	5:7
Visual phototransduction	5:10
G alpha (q) signalling events	4:13
Gastrin-CREB signalling pathway via PKC and MAPK	5:11
G alpha (s) signalling events	4:14
Olfactory Signaling Pathway	5:12
G-protein beta:gamma signalling	4:15
G beta:gamma signalling through BTK	5:13
G beta:gamma signalling through CDC42	5:14
G beta:gamma signalling through PI3Kgamma	5:15
G beta:gamma signalling through PLC beta	5:16
G-protein mediated events	6:2
PLC beta mediated events	7:2
GPCR downstream signalling	3:9
G alpha (12/13) signalling events	4:16
G alpha (z) signalling events	4:17
GPCR ligand binding	3:10
Class C/3 (Metabotropic glutamate/pheromone receptors)	4:20
Hedgehog 'off' state	3:11
Degradation of GLI1 by the proteasome	4:21
GLI3 is processed to GLI3R by the proteasome	4:22
Hedgehog 'on' state	3:12
Activation of SMO	4:23
IGF1R signaling cascade	4:62
IRS-related events triggered by IGF1R	5:45
IRS-mediated signalling	5:38
Insulin receptor signalling cascade	4:51
Intracellular signaling by second messengers	2:2
PIP3 activates AKT signaling	3:4
MAPK family signaling cascades	2:3
MAPK1/MAPK3 signaling	3:5
MAPK6/MAPK4 signaling	3:6
RAF-independent MAPK1/3 activation	4:12
MET promotes cell motility	4:53
MET activates PTK2 signaling	5:39
MET activates RAP1 and RAC1	5:40
Negative regulation of FGFR1 signaling	5:26
Spry regulation of FGF signaling	6:15
Negative regulation of FGFR2 signaling	5:30
Negative regulation of FGFR3 signaling	5:34
Negative regulation of FGFR4 signaling	5:36
Negative regulation of the PI3K/AKT network	4:8
PI5P, PP2A and IER3 Regulate PI3K/AKT Signaling	5:6
P2Y receptors	6:8

DARPP-32 events	6:3
G-protein activation	6:4
Asymmetric localization of PCP proteins	5:50
WNT5A-dependent internalization of FZD2, FZD5 and ROR2	5:51
WNT5A-dependent internalization of FZD4	5:52
AKT phosphorylates targets in the cytosol	4:9
PTEN Regulation	4:10
PKA activation	7:1
Regulation of PTEN gene transcription	5:8
Regulation of PTEN stability and activity	5:9
Chemokine receptors bind chemokines	6:9
Pre-NOTCH Expression and Processing	3:14
Pre-NOTCH Processing in Golgi	4:24
Pre-NOTCH Transcription and Translation	4:25
RHO GTPase Effectors	3:32
RHO GTPases Activate Formins	4:64
RHO GTPases Activate NADPH Oxidases	4:65
RHO GTPases Activate ROCKs	4:66
RHO GTPases Activate WASPs and WAVEs	4:67
RHO GTPases activate CIT	4:68
RHO GTPases activate IQGAPs	4:69
RHO GTPases activate KTN1	4:70
RHO GTPases activate PAKs	4:71
RHO GTPases activate PKNs	4:72
Activated PKN1 stimulates transcription of AR (androgen receptor) regulated genes KLK2 and KLK3	5:48
Signal Transduction	1:1
Integrin signaling	2:4
Signaling by Erythropoietin	2:5
Signaling by GPCR	2:6
Signaling by Hedgehog	2:7
Signaling by Hippo	2:8
Signaling by Leptin	2:9
Signaling by NOTCH	2:10
Signaling by Non-Receptor Tyrosine Kinases	2:11
Signaling by Nuclear Receptors	2:12
Signaling by Receptor Tyrosine Kinases	2:13
Signaling by Rho GTPases	2:14
Signaling by TGF-beta family members	2:15
Signaling by WNT	2:16
mTOR signalling	2:17
Signaling by EGFR	3:21
EGFR downregulation	4:36
GAB1 signalosome	4:37
GRB2 events in EGFR signaling	4:38
SHC1 events in EGFR signaling	4:39
Signaling by ERBB2	3:22
Downregulation of ERBB2 signaling	4:40
ERBB2 Activates PTK6 Signaling	4:31
ERBB2 Regulates Cell Motility	4:41
GRB2 events in ERBB2 signaling	4:42
PI3K events in ERBB2 signaling	4:43

SHC1 events in ERBB2 signaling	4:44
Signaling by ERBB4	3:23
Nuclear signaling by ERBB4	4:45
SHC1 events in ERBB4 signaling	4:46
Erythropoietin activates Phosphoinositide-3-kinase (PI3K)	3:7
Erythropoietin activates RAS	3:8
Signaling by FGFR	3:24
Signaling by FGFR1	4:47
Signaling by FGFR2	4:48
Signaling by FGFR3	4:49
Signaling by FGFR4	4:50
FGFRL1 modulation of FGFR1 signaling	5:27
FGFR2 alternative splicing	5:31
FGFR4 ligand binding and activation	5:37
Hedgehog ligand biogenesis	3:13
Signaling by Insulin receptor	3:25
Insulin receptor recycling	4:52
Signaling by MET	3:26
MET activates RAS signaling	4:54
MET receptor recycling	4:55
Negative regulation of MET activity	4:56
Signaling by NOTCH1	3:15
Signaling by NOTCH3	3:16
Signaling by NOTCH4	3:17
Activated NOTCH1 Transmits Signal to the Nucleus	4:26
NOTCH1 Intracellular Domain Regulates Transcription	4:27
NOTCH3 Activation and Transmission of Signal to the Nucleus	4:28
NOTCH4 Activation and Transmission of Signal to the Nucleus	4:29
Negative regulation of NOTCH4 signaling	4:30
Signaling by NTRK1 (TRKA)	4:57
Nuclear Events (kinase and transcription factor activation)	5:41
Retrograde neurotrophin signalling	5:42
Signalling to ERKs	5:43
Signaling by NTRK2 (TRKB)	4:58
Activated NTRK2 signals through FRS2 and FRS3	5:44
Signaling by NTRKs	3:27
Signaling by NTRK3 (TRKC)	4:59
Signaling by PTK6	3:18
Signaling by Retinoic Acid	3:20
Signaling by PDGF	3:28
Downstream signal transduction	4:60
PTK6 Regulates RHO GTPases, RAS GTPase and MAP kinases	4:32
Signaling by SCF-KIT	3:29
Signaling by Type 1 Insulin-like Growth Factor 1 Receptor (IGF1R)	3:30
Signaling by VEGF	3:31
RA biosynthesis pathway	4:35
Rho GTPase cycle	3:33
Regulation of KIT signaling	4:61
Signaling by TGF-beta Receptor Complex	3:34
TGF-beta receptor signaling activates SMADs	4:73
TGF-beta receptor signaling in EMT (epithelial to mesenchymal transition)	4:74

Signaling by Activin	3:35
Signaling by BMP	3:36
VEGFA-VEGFR2 Pathway	4:63
TCF dependent signaling in response to WNT	3:39
WNT ligand biogenesis and trafficking	3:40
Prolonged ERK activation events	6:30
Signalling to RAS	6:31
p38MAPK events	7:5
Deactivation of the beta-catenin transactivating complex	4:79
Degradation of AXIN	4:80
Degradation of DVL	4:81
Disassembly of the destruction complex and recruitment of AXIN to the membrane	4:82
Formation of the beta-catenin:TCF transactivating complex	4:83
Negative regulation of TCF-dependent signaling by WNT ligand antagonists	4:84
Regulation of FZD by ubiquitination	4:85
Downregulation of TGF-beta receptor signaling	5:49
Regulation of TNFR1 signaling	4:1
TNFR1-induced NFkappaB signaling pathway	4:2
TNFR1-induced proapoptotic signaling	4:3
The phototransduction cascade	6:5
Activation of the phototransduction cascade	7:3
Inactivation, recovery and regulation of the phototransduction cascade	7:4
VEGFR2 mediated cell proliferation	5:46
VEGFR2 mediated vascular permeability	5:47
The canonical retinoid cycle in rods (twilight vision)	6:6
Energy dependent regulation of mTOR by LKB1-AMPK	3:41
mTORC1-mediated signalling	3:42
Regulated proteolysis of p75NTR	4:5
p75NTR signals via NF-kB	4:6
NF-kB is activated and signals survival	5:3
p75NTR recruits signalling complexes	5:4
